# Optimal allocation strategies in platform trials with continuous endpoints

**DOI:** 10.1177/09622802241239008

**Published:** 2024-03-20

**Authors:** Marta Bofill Roig, Ekkehard Glimm, Tobias Mielke, Martin Posch

**Affiliations:** 1 Section for Medical Statistics, Center for Medical Data Science, 27271 Medical University of Vienna, Wien, Austria; 2 Advanced Methodology and Data Science, Novartis Campus, Novartis Pharma AG, Basel, Switzerland; 3 Statistics and Decision Sciences, 60853 Janssen-Cilag GmbH, Neuss, Nordrhein-Westfalen, Germany

**Keywords:** Platform trial, optimal allocation, time trends, shared controls

## Abstract

Platform trials are randomized clinical trials that allow simultaneous comparison of multiple interventions, usually against a common control. Arms to test experimental interventions may enter and leave the platform over time. This implies that the number of experimental intervention arms in the trial may change as the trial progresses. Determining optimal allocation rates to allocate patients to the treatment and control arms in platform trials is challenging because the optimal allocation depends on the number of arms in the platform and the latter typically varies over time. In addition, the optimal allocation depends on the analysis strategy used and the optimality criteria considered. In this article, we derive optimal treatment allocation rates for platform trials with shared controls, assuming that a stratified estimation and a testing procedure based on a regression model are used to adjust for time trends. We consider both, analysis using concurrent controls only as well as analysis methods using concurrent and non-concurrent controls and assume that the total sample size is fixed. The objective function to be minimized is the maximum of the variances of the effect estimators. We show that the optimal solution depends on the entry time of the arms in the trial and, in general, does not correspond to the square root of k allocation rule used in classical multi-arm trials. We illustrate the optimal allocation and evaluate the power and type 1 error rate compared to trials using one-to-one and square root of k allocations by means of a case study.

## 1. Introduction

Platform trials compare multiple experimental treatments to a control. They are multi-arm multi-stage trials with the additional feature of allowing arms to enter and leave the trial over time.^1–4^ As in multi-arm trials, the common trial infrastructure permits shortening the required time and reducing the costs to evaluate new interventions. In addition, the shared control group increases the statistical efficiency compared to separate parallel group trials and requires fewer patients to be allocated to the control group. However, due to the additional flexibility of platform trials, their design and analysis are more complex.

A major concern when designing and analyzing platform trials is the potential presence of time trends, due to, for instance, changes in the patient population being recruited.^5^ Especially, if the allocation rates between each of the active treatment arms and the control group vary over time, such time trends can lead to biased treatment effect estimates and inflation of type I errors in hypothesis tests.^6–9^ This issue has also been discussed for response-adaptive designs.^10^ To address such biases, time period-adjusted analyses based on regression models have been proposed,^11^ where the time periods are defined as the time spans where the allocation ratios stay constant. Simon and Simon^12^ considered randomization-based inference as a solution to avoid the potential bias and to control the type 1 error rate in the presence of time trends.

Time trends are of an even larger concern when the so-called non-concurrent controls are used for treatment-control comparisons. Here, for a specific experimental treatment arm, non-concurrent controls refer to the patients allocated to the control group before the arm under evaluation enters the platform trial. In contrast, concurrent controls are the control group patients randomized concurrently (in time) to those in the treatment arm. While including non-concurrent controls in the estimation of treatment effects can increase the power of testing treatment-control differences and reduce the variance of the estimates, they can also introduce bias in the estimates due to time trends if not adjusted for.^13–16^ Also in this context, time period-adjusted analyses based on regression models have been proposed. They can adjust for potential time trends, and thus control the type 1 error and give unbiased estimates, if time trends in all treatment arms are equal and additive on the model scale.^17^

In this article, we derive optimal treatment allocation rates for platform trials under the assumption that a time period-adjusted analysis based on regression models is used. To understand the principles of optimal allocation strategies in platform trials, we focus on the simple setting of a platform trial with two treatment arms and a shared control, where one of the treatment arms enters when the trial is already ongoing. For this platform trial design, we aim to clarify the design elements on which the optimal allocation ratios depend and to compare the optimal platform trial design with the optimal conventional multi-arm trial design. For the latter, it is well known that for k experimental treatments (and under some additional assumptions), the standard error of treatment effect estimates is minimized for $1:1:\ldots :1:\sqrt{k}$ allocation.^18^

Several authors discuss the problem of adding a new treatment arm during the ongoing trial using different optimality criteria and statistical analysis procedures. Cohen et al.^19^ reviewed statistical methodologies and examples of trials with newly added treatment arms. Choodari-Oskooei et al.^20^ focused on the family wise type 1 error rate when new arms are added. Elm et al.^21^ evaluated the operating characteristics of pairwise comparisons of trials adding a new arm over the trial under different approaches. Ren et al.^22^ described statistical considerations with respect to type 1 error and power in three-arm umbrella trials. They also discussed the optimal allocation ratio for the control arm in periods in which treatment arms overlap, when minimizing the sum of variances of the treatment effect estimators. However, to the best of our knowledge, Bennett and Mander^23^ is the first article in which the optimal allocation rates in platform trials are investigated. They optimized the allocation rates to maximize the probability to find all treatments that are better than the control, while assuming that the expected treatment effects were equal for all treatment arms. In their approach, treatment comparisons are based on simple group comparisons with z -tests, using concurrent controls. Concurrent data from different periods (where other treatments may have entered or left the platform and the allocation ratios may have changed) are pooled in this approach. However, in platform trials with time trends and changing allocation ratios over time, this approach can lead to an inflation of the type 1 error rate and biased estimates.^12^ More recently, Pan et al.^24^ addressed the modification of the critical boundaries to control the family wise error rate and re-estimation of the sample sizes when new arms are added, and, similarly as by Bennett and Mander,^23^ provided the optimal allocation ratios when minimizing the total sample size to achieve a desirable marginal power level, using only concurrent controls and without adjusting for potential time trends.

Here, we optimize allocation rates for a testing procedure based on a regression approach, which includes a “period” effect to account for changing allocation ratios when using period-wise treatment effect estimators and focus the attention on the marginal power. We also consider a different optimization criterion. Instead of the power to reject all null hypotheses corresponding to effective treatments, we minimize the maximum of the standard errors of the means of the treatment effect estimators resulting from the regression model. Assuming equal targeted treatment effects, this is asymptotically equivalent to maximizing the minimum power across the investigated treatments. This implies (under some regularity assumptions) that, under the optimal design, the power of the different arms will be equal. In particular, for multi-sponsor platform trials, this is a reasonable feature, as all sponsors should get the same chance to demonstrate the efficacy of their treatments in the platform. In addition, besides platform trials that only use concurrent data to compare treatments against control, we consider trials that incorporate non-concurrent controls.

The article is structured as follows. In Section 2, we introduce the platform trial designs considered and introduce the notation. In Section 3, we derive optimal allocations for trials using concurrent controls only and, in Section 4, do so for trials also incorporating non-concurrent controls. In Section 5, we illustrate the application of different allocation rules (optimal compared to equal allocation and square root of k allocation) by a simulation study based on an example in the context of a hypercholesterolemia trial. In Section 6, we discuss optimal allocations under unequal variances (Section 6.1) and optimal allocations when minimizing the sum of the variances (Section 6.2). We conclude the article with a discussion.

## 2. Trial designs and optimality criterion

Consider a platform trial evaluating the efficacy of two experimental arms ( i=1,2 ) against a shared control ( i=0 ). The trial design allows the sequential entry and exit of the experimental arms, such that the trial initially starts with arm 1 and the control, and arm 2 may enter the platform trial at a later time point. In addition, recruitment to arm 1 may end before the recruitment to arm 2 ends. Thus, the platform trial consists of three periods ( s=1,2,3 ) defined according to the sets of actively recruiting treatment arms $I_{s}$ . In period 1, patients are recruited to treatment 1 and control ( $I_{1}=\{0,1\}$ ), in period 2 to both experimental treatments and control ( $I_{2}=\{0,1,2\}$ ) and, in period 3, to treatment 2 and control ( $I_{3}=\{0,2\}$ ).

**Figure 1.**
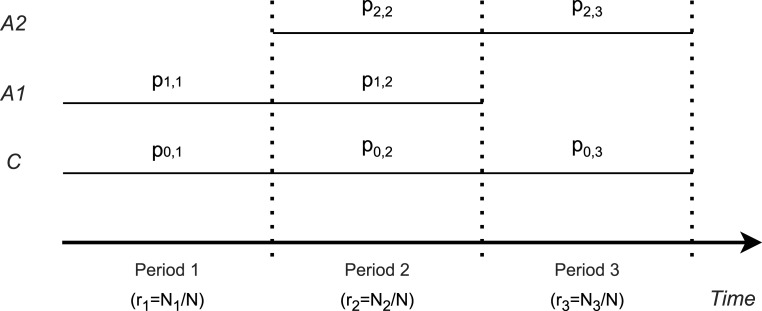
Platform trial with two active treatments ( $A_{1}$ and $A_{2}$ ) and a shared control ( C ). The trial duration is divided into three periods. $p_{i,s}$ denotes the allocation ratios in period s ( s=1,2,3 ) to arm i ( i=0,1,2 ), $r_{s}=N_{s}/N$ refers to the proportion of patients in period s , where $N_{s}$ and N are the sample sizes in period s and in the overall trial, respectively.

The total sample size of the trial is denoted by N , which is partitioned into three periods with sample sizes $N_{s}$ , s=1,2,3 . We refer to the corresponding proportions of patients by $r_{s}=N_{s}/N$ . In each period s , patients are allocated to the arms $i\in I_{s}$ with the allocation ratios $p_{i,s}$ , such that $\sum_{i\in I_{s}}p_{i,s}=1$ for s=1,2,3 . See Figure 1 for an illustration of the trial design.

Let $y_{j}$ denote the observation of patient j on treatment arm $i_{j}$ ( j=1,…,N , $i_{j}=0,1,2$ ), distributed as $y_{j}∼N(\mu_{i},\sigma^{2})$ where σ is assumed to be known and where we dropped the subindex j in the mean for simplicity. Let $\theta_{i}=\mu_{i}-\mu_{0}$ denote the treatment effect for treatment i ( i=1,2 ), and consider the null hypotheses $H_{i}:\theta_{i}\leq 0$ .

The simple mean differences between the treatment and control groups are biased estimators of the treatment effects if there are time trends (i.e. the means in the treatment arms change over time) and the allocation rates change across periods.^5^ Therefore, we use stratified estimators, stratified by period, that adjust for potential time trends.^9,25,26^ The stratified estimators are given by

(1) $${\hat{\theta }}_{i}=\sum_{s=i,i+1}w_{i,s}\cdot {\hat{\theta }}_{i,s}$$

where ${\hat{\theta }}_{i,s}={\bar{y}}_{i,s}-{\bar{y}}_{0,s}$ is the treatment effect estimate in period s for arm i ( i=1,2 ), where ${\bar{y}}_{i,s}$ and ${\bar{y}}_{0,s}$ are the sample means in period s for experimental and control arms, and the weights are given by

(2) $$w_{1,s}=\frac{\frac{1}{\sigma_{1,s}^{2}}}{\frac{1}{\sigma_{1,1}^{2}}+\frac{1}{\sigma_{1,2}^{2}}}\;\;\text{and}\;\;w_{2,s}=\frac{\frac{1}{\sigma_{2,s}^{2}}}{\frac{1}{\sigma_{2,2}^{2}}+\frac{1}{\sigma_{2,3}^{2}}}$$

where $\sigma_{i,s}^{2}=\mathrm{Var}({\hat{\theta }}_{i,s})$ denotes the variances of the estimates per period, and is given by $\sigma_{i,s}^{2}=\sigma^{2}(1/n_{i,s}+1/n_{0,s})$ . This choice of weights minimizes the variance of the stratified treatment effect estimator. Then, the variance of the stratified effect estimator is given by

(3) $$\mathrm{Var}({\hat{\theta }}_{i})=\sum_{s=i,i+1}w_{i,s}^{2}\sigma_{i,s}^{2}=\frac{\sigma^{2}}{N}\cdot {\left(r_{i}\frac{p_{i,i}p_{0,i}}{p_{i,i}+p_{0,i}}+r_{i+1}\frac{p_{i,i+1}p_{0,i+1}}{p_{i,i+1}+p_{0,i+1}}\right)}^{-1}$$

A few comments: (i) The stratified estimator is equivalent to the non-stratified estimator if the allocation ratios are equal across periods. (ii) Expression (3) for the variances applies if the above distributional assumptions hold, that is, if there are no time trends. However, as discussed above, the stratified treatment effect estimate used is also unbiased if time trends are present. (iii) The stratified estimator (and corresponding test) corresponds to the treatment effect estimators in the linear models:

(4) $$\begin{array}{rl} E(y_{j}) & =\eta_{1}+\theta_{1}\cdot 1(i_{j}=1)+\theta_{2}\cdot 1(i_{j}=2)+\tau_{2}\cdot 1(j\in (N_{1}+1,N_{2}]) \end{array}$$

(5) $$\begin{array}{rl} E(y_{j}) & =\eta_{2}+\theta_{1}\cdot 1(i_{j}=1)+\theta_{2}\cdot 1(i_{j}=2)+\tau_{3}\cdot 1(j\in (N_{2}+1,N]) \end{array}$$

where 1(⋅) denotes the indicator function. The first model (to test $H_{1}$ ) is fit with observations from periods 1 and 2 of the control and treatment arms 1 and 2 and the second model (to test $H_{2}$ ) with observations from periods 2 and 3 of the control and arms 1 and 2. Here $\tau_{s}$ denotes the period effect which adjusts for potential time trends. The treatment effects are assumed to be constant in time (no time-by-treatment interaction).

The optimality criterion we aim to minimize is the maximum of the variances of the stratified effect estimators across experimental treatment arms. Thus, given a fixed overall sample size N , we aim to find the allocation probabilities $p=(p_{0,1},p_{1,1},p_{0,2},p_{1,2},p_{2,2},p_{0,3},p_{2,3})$ that minimize the objective function

(6) $$\max(\mathrm{Var}({\hat{\theta }}_{1}),\mathrm{Var}({\hat{\theta }}_{2}))$$

If there is no unique solution to this optimization problem, we aim to find the solution leading to the smallest minimum variance $\min(\mathrm{Var}({\hat{\theta }}_{1}),\mathrm{Var}({\hat{\theta }}_{2}))$ among all allocation probabilities leading to the same optimized maximum variance (6).

Note that if the expected effect sizes are equal for both experimental arms, minimizing (6) will also maximize the minimum individual power. Furthermore, as N is only a scaling factor considered fixed in (3), the optimal allocation does not depend on N . Similarly, assuming $\sigma^{2}$ to be known is not a limitation, because the optimal allocation does not depend on the variances, as long as they are equal across arms. The optimization under unequal variances will be discussed in Section 6.1. Finally, as mentioned above, the analysis adjusts for biases due to time trends, but optimization is performed under the assumption that there is no time trend. This is because at the planning stage it is usually unknown whether time trends will be present in the trial or what pattern they will follow.

## 3. Optimal designs

We derive optimal designs minimizing the objective function (6) for three different cases:

Case 1: **Unrestricted optimization.** In this setting, the entry time for treatment 2 (corresponding to $r_{1}$ ) is a design parameter that is determined by the trial design rather than being externally governed, for example, by the time a new treatment becomes available to be included in the trial. Similarly, the time from entry of treatment 2 and completion of the sub-trial corresponding to treatment 1, $r_{2}$ , is a design parameter in this case, which can also be chosen. We therefore optimize over p and $r_{1},r_{2}$ ( $r_{3}$ is then given by $r_{3}=1-r_{1}-r_{2}$ ).
Case 2: **Fixed sample size in period 1.** We fix $r_{1}$ and optimize p and $r_{2}$ . In this case, the entry time of arm 2 is not subject to optimization.
Case 3: **Fixed sample sizes in all three periods.** In addition to $r_{1}$ , we also fix $r_{2}$ (and thus also $r_{3}$ ). Therefore, this case corresponds to a design in which both the entry time of arm 2 ( $r_{1}$ ) and the completion time of arm 1 (given by $r_{1}+r_{2}$ ) are predefined, and we optimize over p only.

The three cases differ with regard to the parameters to be optimized. In Case 3, the proportions of patients in periods 1, 2, and 3 are fixed, and the allocation ratios in each period are optimized. In Case 2, only the proportion of patients in period 1 is fixed, and, in addition to the allocation ratios within periods, the proportion of patients recruited in period 2 is optimized. Finally, in Case 1, the proportion of patients recruited in periods 1, 2, and 3 and the allocation ratios within the period are optimized. Assuming a constant recruitment rate, the proportions of patients in the different periods correspond to the entry and exit times of the experimental treatment arms. Instead of assuming the times to be deterministic, one could consider the input times to be random. This consideration is discussed in Section 7. As we will see, the three cases give qualitatively different optimal designs, which differ in the number of periods. Table 1 provides a summary of the design parameters that are assumed to be fixed and the parameters that are optimized, and depicts the structure of the optimal solution for each case.

**Table 1.** Summary of considered designs and optimal solutions.

	Design assumptions	Optimized parameters	Solution
Case 1	(None)	$r_{1}$ , $r_{2}$ , p	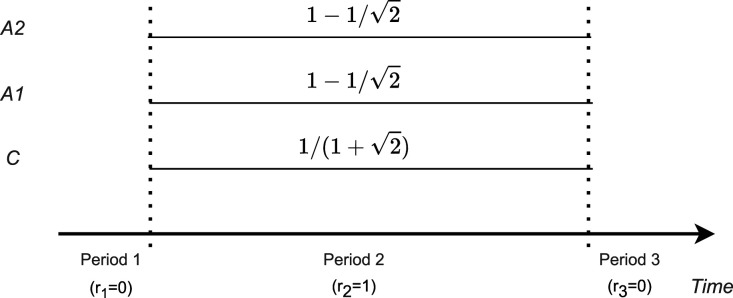
Case 2	∙ Fixed entry time $A_{2}$	$r_{2}$ , p	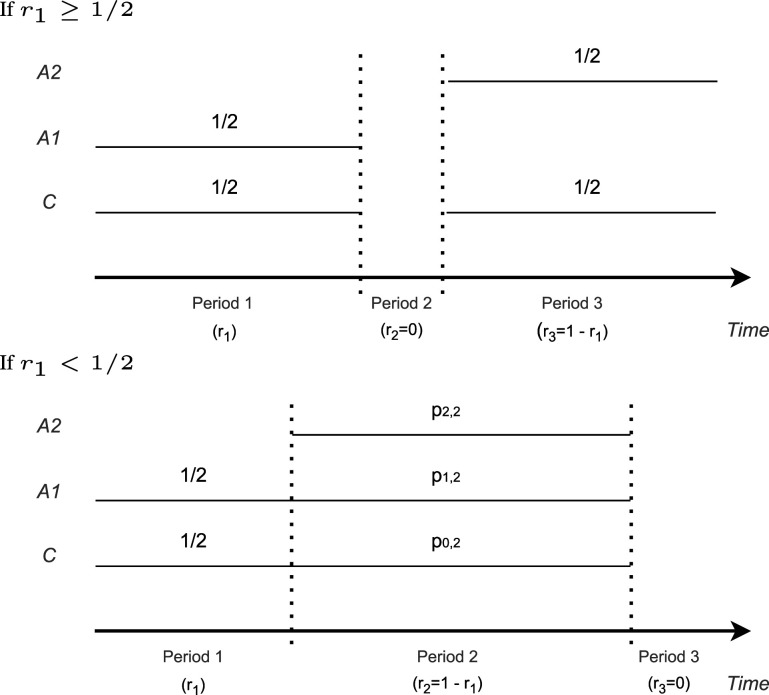
Case 3	∙ Fixed entry time $A_{2}$ ∙ Fixed exit time $A_{1}$	p	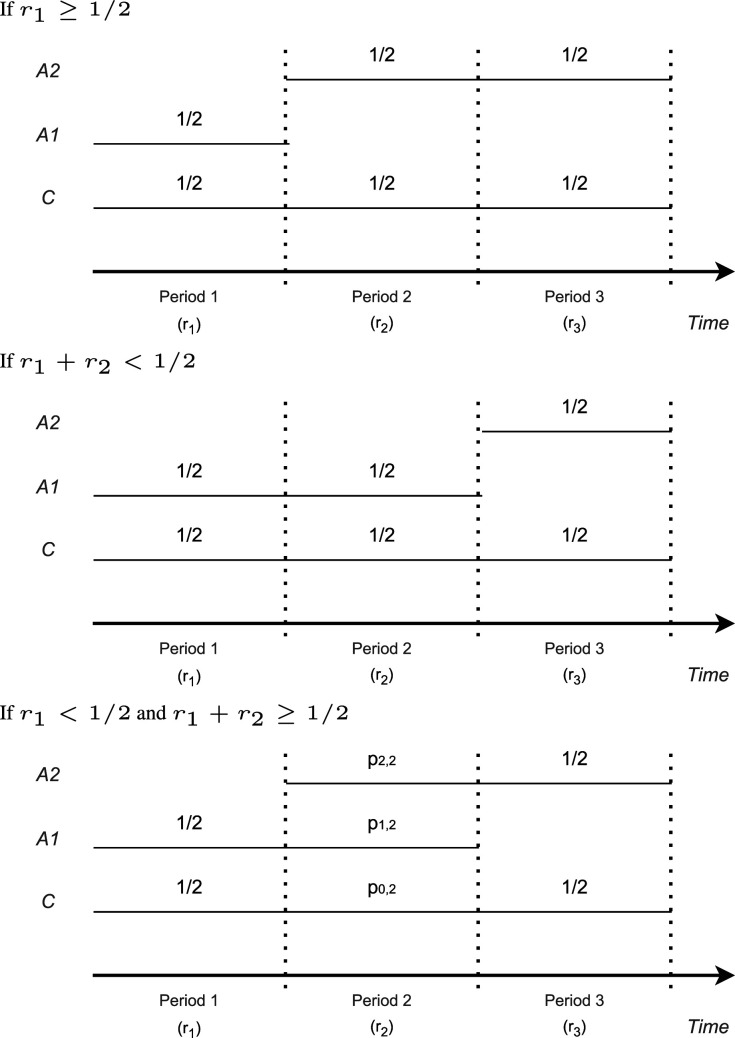

The first column indicates the case (see Section 3), the second specifies the assumptions regarding the entry and exit times of arms in the trial, the third lists the optimized parameters, and the fourth column illustrates the optimal design. The solutions for the allocation ratios in period 2 in Case 2 with $r_{1}<1/2$ and Case 3 with $r_{1}<1/2$ and $r_{1}+r_{2}\geq 1/2$ can be found in Sections 3.2 and 3.3 for concurrent controls, and Sections 4.2 and 4.3 for non-concurrent controls. The code to obtain the solutions is shown in Supplemental Material D.

In the subsections below, we present the derivations of the optimal allocations in each of the three cases. In the derivations, we assume that the sample sizes are positive real numbers such that the variance of the estimators (3) is a differentiable function of the sample sizes. This is a reasonable approximation for large sample sizes. The derivations were performed in Mathematica (see Section D in the Supplemental Material, and GitHub (https://github.com/MartaBofillRoig/Allocation). In calculations resulting in several solutions, we selected those resulting in real values between 0 and 1 based on numeric examples only.

Furthermore, since stratified test statistics are used, the optimal allocation in periods 1 and 3 is equal allocation, that is, $p_{0,1}=p_{1,1}=p_{0,3}=p_{2,3}=1/2$ . This can be directly seen from equation (3): the allocation ratios in period 1 only affect $Var({\hat{\theta }}_{1})$ and, since $p_{0,1}=1-p_{1,1}$ , it follows that $p_{1,1}p_{0,1}/(p_{1,1}+p_{0,1})$ is maximized for $p_{1,1}=p_{0,1}=1/2$ . A similar argument holds for the optimal allocation in period 3. Thus, it remains to determine the optimal allocation in period 2 and, depending on the case considered, the optimal partition between periods by means of $r_{s}$ .

Note that if the optimal allocation in period 2 allocates positive proportions of subjects to both experimental arms (i.e. if $p_{12},p_{22}\neq 0,1$ ) then the optimal allocation satisfies the constraint

(7) $$Var({\hat{\theta }}_{1})=Var({\hat{\theta }}_{2})$$

This follows by the following argument: Assume the variances at the optimal allocation are not equal, for example, that $Var({\hat{\theta }}_{1})<Var({\hat{\theta }}_{2})$ . Then, the objective function can be further decreased by moving a small fraction of the sample size from treatment 1 to treatment 2 such that the inequality still holds. This shift reduces the $Var({\hat{\theta }}_{2})$ and thereby the maximum of the two variances. This is a contradiction to the assumption that the allocation is optimal.

### 3.1. Case 1: Unrestricted optimization

In this case, the optimal design satisfies $r_{2}=1$ (and $r_{1}=r_{3}=0$ ) such that all patients are recruited in period 2. In this design, there is only a single period and the effect estimates reduce to the non-stratified mean differences. The one-period design is optimal because then all control observations are shared and are used in both treatment effect estimates, and a non-stratified estimate is applied which, under the model assumption of no time trend, also reduces the variances. The resulting trial is a classical multi-armed trial with many-to-one comparisons.^18^ For this design, it is well known that the optimal allocation is $p_{0,2}=1/(1+\sqrt{2})$ , $p_{1,2}=p_{2,2}=1-1/\sqrt{2}$ .

### 3.2. Case 2: Fixed sample size in period 1

Assume that the time point when the second treatment enters the platform trial is given. Thus, the size of period 1 ( $r_{1}$ ) is fixed and we optimize (6) over $r_{2}$ and p . As noted above, the optimal allocation in the first and third periods is equal allocation. We derive the optimal allocation in period 2 for all $r_{1}\in (0,1)$ , considering first the scenarios where $r_{1}\geq 1/2$ and then the scenarios where $r_{1}<1/2$ .

If $r_{1}\geq 1/2$ , period 1 is larger than or equal to half the total sample size. Therefore, for any choice of allocation ratios within periods 2 and 3, we have $Var({\hat{\theta }}_{2})\geq Var({\hat{\theta }}_{1})$ (where equality can hold only for $r_{1}=1/2$ ), even if all observations in periods 2 and 3 are allocated to arm 2 and the control. As the objective function is the maximum of the two variances, it is minimized in this case, if all patients $j>N_{1}$ are equally allocated to treatment 2 and control, that is, for $p_{0,1}=p_{1,1}=1/2$ , $p_{0,3}=p_{2,3}=1/2$ , and $r_{2}=0$ . This optimal solution corresponds to two separate consecutive trials, wherein the first period we compare arm 1 versus control, there is no second period, and in the third period, we use the remaining sample size to compare arm 2 versus control. Note that all designs with $0<r_{2}\leq 1-r_{1}$ , where all patients are allocated either to arms 0 and 2 in periods 2 and 3, minimize $Var({\hat{\theta }}_{2})$ as well, and are therefore also optimal solutions. Moreover, if $Var({\hat{\theta }}_{2})>Var({\hat{\theta }}_{1})$ under optimal allocation (as is the case for $r_{1}<1/2$ ), the maximum variance does not change if the allocation ratios in period 1 slightly deviate from equal allocation. Therefore, the optimal design is not unique when the objective function is just the maximum of the variances. However, with the additional condition of the smallest minimum variance among all minmax solutions (see the condition below (6)) equal allocation in the first period is optimal.

Otherwise, if $r_{1}<1/2$ , one can see that under the optimal design in period 2, patients are allocated to both experimental treatment arms. Therefore, as discussed at the beginning of Section 3, under the optimal design satisfies the variances of the effect estimators are equal (7). Furthermore, for the optimal design we have $r_{2}=1-r_{1}$ and $r_{3}=0$ . This can be confirmed by numerical optimization. Figure 3 shows the maximum variance under optimal allocation compared to the maximum variance of optimized separate trials with the same total sample size, as a function of $r_{2}$ . One can see that the maximum variance is minimized for $r_{2}=1-r_{1}$ . This is due to the following argument: If the period 3 observations in arms 2 and control are all moved to the respective arms in period 2, both, the variances of arms 1 and 2 will decrease. For arm 1, this holds because the concurrent control group sample size increases. For arm 2, this is the case because a non-stratified estimator is used instead of a stratified estimate, the non-stratified estimator having lower variance under the model assumption of no time trend.

The optimal allocations $p_{0,2}$ , $p_{1,2}$ , and $p_{2,2}$ can be obtained numerically as a special case of Case 3, setting $r_{2}=1-r_{1}$ , outlined below. Hence, the optimal design is a two-period trial in which arm 2 enters later, but both arms finish at the end of the trial. In this case, the numerical examples suggest that the optimal allocation is unique.

### 3.3. Case 3: Fixed sample sizes in all three periods

Assume that, in addition to $r_{1}$ , the proportion of recruited patients when treatment 2 enters, also $r_{2}$ , the proportion of patients in period 2 is fixed. To derive the optimal allocation in period 2, we distinguish three scenarios: (i) $r_{1}\geq 1/2$ ; and, for $r_{1}<1/2$ : (ii) $r_{1}+r_{2}<1/2$ , and (iii) $r_{1}<1/2$ and $r_{1}+r_{2}\geq 1/2$ .

In scenario (i), if $r_{1}\geq 1/2$ , as in Case 2, the optimal allocation assigns all patients after period 1 to treatment arm 2 and control, with equal allocation between the two arms. This is achieved, for example, for $p_{0,1}=p_{1,1}=p_{0,2}=p_{2,2}=p_{0,3}=p_{2,3}=1/2$ and $p_{1,2}=0$ . With the optimality condition, to achieve the smallest minimum variance among all minmax solutions (see the condition (6)), equal allocation in period 3 is optimal.

In scenario (ii), if $r_{1}+r_{2}<1/2$ , then $r_{1}<1/2$ and $r_{3}\geq 1/2$ . Analogously to scenario (i), the optimal design allocates patients in period 2 to treatment arm 1 and control such that $p_{0,2}=p_{1,2}=1/2$ , $p_{2,2}=0$ . This design minimizes the variance of ${\hat{\theta }}_{1}$ , which is the maximum of the variances in this case.

In scenario (iii) if, on the other hand, $r_{1}<1/2$ and $r_{1}+r_{2}\geq 1/2$ , one can see that under the optimal design, patients are allocated to both experimental treatment arms in period 2. Therefore, as discussed at the beginning of Section 3, the variances of the two treatment effect estimators are equal under optimal allocation, cf. equation (7). To optimize under this constraint, we use the method of Lagrange multipliers, which shows that the solution satisfies

(8) $$\begin{array}{rl} p_{0,2} & =\frac{1}{2(1-p_{2,2})}-p_{2,2} \end{array}$$

(9) $$\begin{array}{rl} \frac{r_{2}}{1-2r_{1}} & =\frac{(1-p_{2,2}{)}^{3}}{\left(2p_{2,2}-1)(p_{2,2}(p_{2,2}(p_{2,2}(2p_{2,2}(2p_{2,2}-7)+19)-15)+7)-2\right)} \end{array}$$

Now, $p_{2,2}$ can be obtained as the numerical solution of (9) and $p_{0,2}$ is given by (8). As the sum of the allocation ratios is 1 also $p_{1,2}$ results. Note that by (9) that the optimal solution depends on $r_{1},r_{2}$ only via $r_{2}/(1-2r_{1})$ . The numerical examples suggest that the optimal allocation is unique in this case.

**Figure 2.**
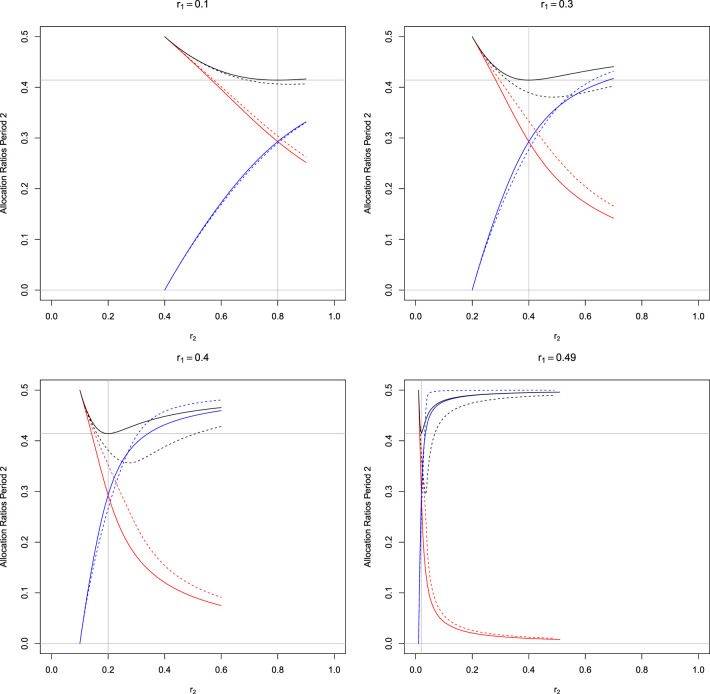
Optimal allocation probabilities $p_{i,2},i=0,1,2$ in period 2 as function of $r_{2}$ and for different values of $r_{1}$ in trials with three-periods as shown in Figure 1. Black lines: $p_{0,2}$ , red line: $p_{1,2}$ , and blue line: $p_{2,2}$ . The vertical line at $r_{2}=1-2*r_{1}$ indicates where equal allocation between treatments 1 and 2 is optimal for the design with concurrent controls only. The horizontal line marks the value $1/(1+\sqrt{2})$ . Solid lines correspond to the optimal allocations in trials utilizing concurrent controls only (Section 3), and dashed lines correspond to the optimal allocations in trials utilizing concurrent and non-concurrent controls (Section 4).

Figure 2 shows the optimal allocation probabilities $p_{i,2},i=0,1,2$ in period 2 as functions of $r_{2}$ for different $r_{1}$ . The optimal allocation ratio $p_{0,2}$ for the control is not monotone in $r_{2}$ but has a minimum at $r_{2}=1-2r_{1}$ , that is, where $r_{1}=r_{3}$ . For this special case, we can derive an explicit solution for the optimization problem. As in this case, the objective function is symmetric in the two treatments, it follows that $Var({\hat{\theta }}_{1,1})=Var({\hat{\theta }}_{2,3})$ . Therefore, to satisfy constraint (7), also $Var({\hat{\theta }}_{1,2})=Var({\hat{\theta }}_{2,2})$ needs to hold. The latter, however, implies that in period 2, the allocation ratios for both treatment arms have to be equal, such that $p_{1,2}=p_{2,2}$ . Furthermore, by results for the many-to-one setting,^18^ we know that the variances are minimized for $1:1:\sqrt{2}$ allocation, such that $p_{0,2}=1/(1+\sqrt{2})$ and $p_{1,2}=p_{2,2}=1-1/\sqrt{2}$ . This can be also verified by substituting the solution in (9).

**Figure 3.**
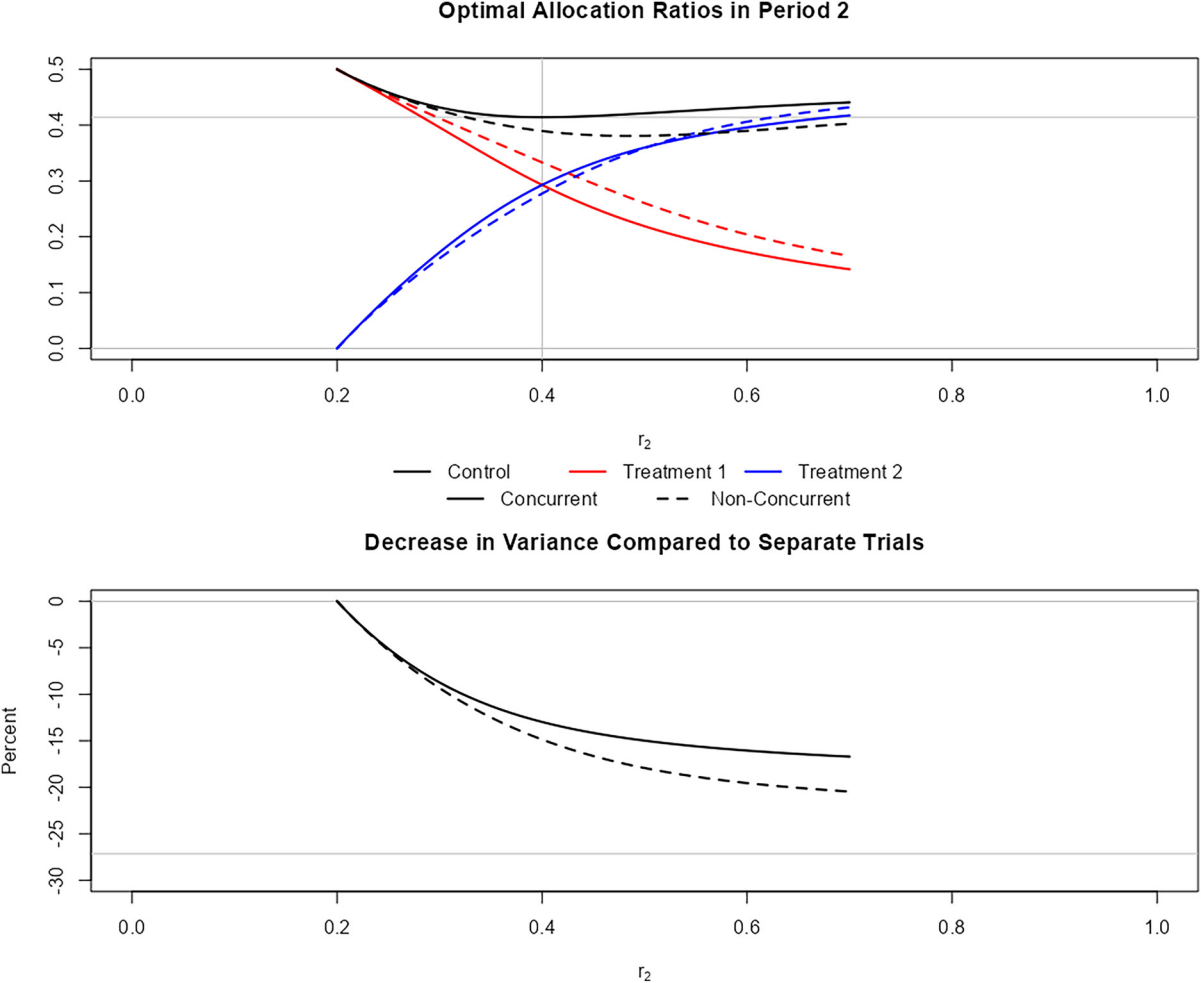
Screenshot from the shiny app (available at https://github.com/MartaBofillRoig/Allocation). The figure on the top shows the optimal solutions for $r_{1}=0.3$ as shown in Figure 2. The figure on the bottom compares the maximum of the two variances to the variance using separate trials. Solid lines indicate the solutions for trial designs using concurrent controls only, while dashed lines correspond to those for trials using concurrent and non-concurrent controls.

In order to illustrate further cases apart from those included in the article, we built a shiny app (available at https://github.com/MartaBofillRoig/Allocation) that allows visualizing the allocation rates with respect to the sample sizes per period 2. The app also shows the variance in the optimum as a function of $r_{2}$ . Figure 3 includes one of the figures that can be obtained in the app. On the bottom, we see the variance with respect to the proportion of $r_{2}=N_{2}/N$ compared to the variance when running two separate trials. We can observe that the decrease in variance becomes larger with $r_{2}$ .

## 4. Optimal allocation in trials using non-concurrent controls

Suppose we want to use also non-concurrent controls to compare arm 2 against the control. To this end, we consider the regression model estimate of the treatment effect of treatment 2 from the model

(10) $$E(y_{j})=\eta_{0}+\sum_{i=1,2}\theta_{i}\cdot 1(i_{j}=i)+\sum_{s=2,3}\tau_{s}\cdot 1(j\in (N_{s-1}+1,N_{s}])$$

We fit this model based on all observed data in periods 1, 2, and 3. This model provides unbiased treatment effect estimators if time trends affect all arms equally and are additive on the model scale.^17^ For treatment 1, we suppose that the efficacy is evaluated after treatment arm 1 ends, as is common in platform trials. Thus, we do not use the estimate from this model, but the stratified treatment effect estimators defined in (4) based on data from treatment 1 and the control group from stages 1 and 2 only.

Using the regression model (10) to incorporate non-concurrent controls implies that the treatment effect of arm 2 compared to control is estimated by the stratified treatment effect estimator

(11) $${\tilde{\theta }}_{2}=\omega_{1,1}({\bar{y}}_{1,1}-{\bar{y}}_{.,1})+\omega_{1,2}({\bar{y}}_{1,2}-{\bar{y}}_{.,2})+\omega_{2,2}({\bar{y}}_{2,2}-{\bar{y}}_{.,2})+\omega_{2,3}({\bar{y}}_{2,3}-{\bar{y}}_{.,3})$$

where ${\bar{y}}_{.,s}$ is the pooled mean per period, and $\omega_{i,s}$ are weights, which are functions of the sample sizes. As a consequence, also the variance of ${\tilde{\theta }}_{2}$ can be written as a function of the allocation rates p and the total sample size N . This variance is given by

(12) $$\mathrm{Var}({\tilde{\theta }}_{2})=\frac{\sigma^{2}}{N}{\left(r_{3}p_{2,3}(1-p_{2,3})+r_{2}\left(p_{2,2}(1-p_{2,2})-\frac{r_{2}p_{1,2}^{2}p_{2,2}^{2}}{r_{1}p_{1,1}(1-p_{1,1})+r_{2}p_{1,2}(1-p_{1,2})}\right)\right)}^{-1}$$

See Section A2 in the Supplemental Material for the derivation and the expression of the weights $\omega_{i,s}$ . For arm 1, for which the treatment effect is estimated using only concurrent controls, the treatment effect estimator and its variance are given by (1) and (3), respectively.

As in Section 3, we aim to minimize the maximum of the variances $\max(\mathrm{Var}({\hat{\theta }}_{1}),\mathrm{Var}({\tilde{\theta }}_{2}))$ in p and discuss the optimal allocations for unrestricted optimization (Case 1), where the entry time of treatment 2 is fixed (Case 2) and where both the entry time of treatment 2 and the completion time of treatment 1 are given (Case 3).

It is evident from (3) and (12) that for all $r_{1},r_{2},r_{3}$ (and thus for all three considered cases), the optimal allocation in periods 1 and 3 is equal allocation, that is, $p_{0,1}=p_{1,1}=p_{0,3}=p_{2,3}=1/2$ . This is due to the fact that we consider a stratified estimator. Therefore, it remains to determine the optimal allocation ratios in period 2 in the three cases.

### 4.1. Case 1: Unrestricted optimization

As for the setting using concurrent controls only, without restrictions with respect to the time at which arm 2 enters the platform, the optimal allocation rates correspond to a multi-arm design where all treatment arms start and end at the same time, such that there are no non-concurrent controls. This follows because then all control patients are used in direct comparisons for both treatment arms. Then, the optimal allocation is $1:1:\sqrt{2}$ allocation as in Section 3.

### 4.2. Case 2: Fixed sample size in period 1

Suppose we aim at optimizing a design where the time at which arm 2 enters is given. Therefore, as in Case 2 for trials using concurrent controls only (Section 3.2), we optimize assuming that $r_{1}$ is fixed.

As for concurrent controls, we derive the optimal allocation, considering first scenarios where $r_{1}\geq 1/2$ and then scenarios where $r_{1}<1/2$ . If $r_{1}\geq 1/2$ , as in the case of concurrent controls, the optimal allocation allocates all patients after period 1 to treatment arm 2 and control, with equal allocation between the two arms. This is achieved, for example, for $p_{0,1}=p_{1,1}=1/2$ , $p_{0,3}=p_{2,3}=1/2$ , and $p_{i,2}=0$ , i=0,1,2 such that $r_{2}=0$ . Note that this design has only two periods and the model (10) is fitted without the period effect $\tau_{2}$ as there is no data to estimate this factor in this case. In addition, the non-concurrent controls do not contribute to the treatment effect estimator (only the weight $\omega_{2,3}$ in (11) is larger than zero) because only the control treatment is present in both periods. Therefore, the period effect can only be estimated from the control group data and consequently the estimate of the control group treatment (and also the treatment effect estimate for treatment 2) cannot be improved by estimates from other treatments.

To see that the above allocation is optimal, note that $Var({\tilde{\theta }}_{2})$ defined in (12) is minimized by $r_{3}=1-r_{1},r_{2}=0$ , and $p_{0,3}=p_{2,3}=1/2$ if $r_{1}$ is kept fixed and for this allocation the variance $Var({\tilde{\theta }}_{2})$ does not depend on the allocation ratios in period 1. Furthermore, because $r_{1}\geq 1/2$ and assuming equal allocation between the control arm and arm 1 in period 1, we have $Var({\hat{\theta }}_{1})\leq Var({\tilde{\theta }}_{2})$ . Therefore, under an allocation ratio that minimizes $Var({\tilde{\theta }}_{2})$ the objective function, the maximum of the variances of the treatment effect estimates is the variance of treatment 2. It follows, that this allocation ratio also minimizes the objective function.

If $r_{1}<1/2$ , as in Case 2 in Section 3, the optimal design satisfies that $r_{2}=1-r_{1}$ , and therefore leads to a two-period platform trial, where treatments 1 and 2 complete recruitment at the same time and there is no period 3. While this can be seen by inspection of Figure 3, there is also an argument for this result. First, note that for any given three-period platform trial (with $r_{2},r_{3}>0$ ), we can achieve the same sample sizes for each arm in a two-period platform trial, shifting all observations from period 3 to period 2 such that $r_{3}=0$ . Now, after period 1, recruiting all observations of the control arm in period 2 instead of splitting them between periods 2 and 3, increases the sample size of the control arm for the estimate of ${\hat{\theta }}_{1}$ and, therefore, reduces its variance. Also, the variance of ${\tilde{\theta }}_{2}$ is not increased, as the number of concurrent controls for arm 2 is not affected and, as $r_{3}=0$ , the model (10) is fitted with one parameter less (without $\tau_{3}$ ) in this case.

It remains to determine the optimal allocation in period 2 for the resulting two-period platform trial. In this case, the effect size estimate of treatment 2 using non-concurrent controls (11) can be written as

(13) $${\tilde{\theta }}_{2}={\hat{\theta }}_{2,2}+\rho ({\hat{\theta }}_{1,1}-{\hat{\theta }}_{1,2})$$

where $\rho =n_{0,2}^{-1}/(n_{0,1}^{-1}+n_{0,2}^{-1}+n_{1,1}^{-1}+n_{1,2}^{-1})$ and its variance simplifies to

(14) $$\mathrm{Var}({\tilde{\theta }}_{2})=\frac{\sigma^{2}}{N}{\left(r_{2}q_{2,2}-\frac{r_{2}^{2}p_{1,2}^{2}p_{2,2}^{2}}{r_{1}q_{1,1}+r_{2}q_{1,2}}\right)}^{-1}$$

where $q_{i,s}=p_{i,s}(1-p_{i,s})$ .

We now minimize $\max(\mathrm{Var}({\hat{\theta }}_{1}),\mathrm{Var}({\tilde{\theta }}_{2}))$ , where $\mathrm{Var}({\hat{\theta }}_{1})$ and $\mathrm{Var}({\tilde{\theta }}_{2})$ are defined by (3) and (14), respectively. For given $r_{1}$ and assuming that $r_{3}=0$ , with a similar argument as at the beginning of Section 3, one can see that (7) holds, and the variances under the optimal allocation are equal. Using this constraint, we obtain an explicit expression for the optimal allocation by minimizing $\mathrm{Var}({\hat{\theta }}_{1})$ setting $p_{01}=p_{11}=1/2$ . The resulting formulas for the allocation ratios in period 2 are to be found in the Appendix.

**Figure 4.**
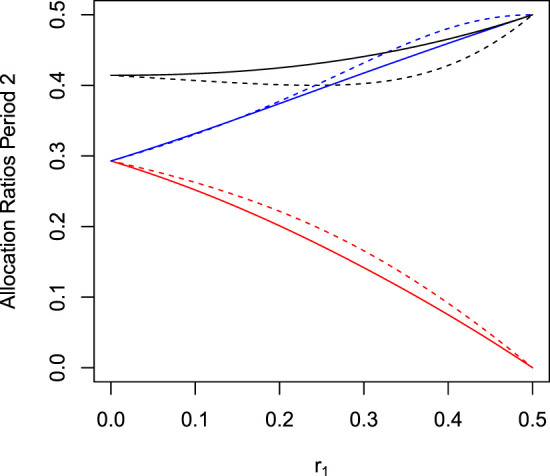
Optimal allocation probabilities $p_{i,2}$ , i=0,1 in period 2 as function of $r_{1}$ for trials with two-periods ( $r_{3}=0$ ). Black, red, and blue lines represent the allocation rates to control, arm 1, and arm 2, respectively. Solid lines refer to analysis with concurrent controls only, and dashed lines to analysis with concurrent and non-concurrent controls.

Figure 4 displays the optimal allocations as a function of $r_{1}$ both, for trials using non-concurrent controls (dashed lines) and using only concurrent controls (solid lines). Comparing the period 2 allocation rates of the optimal designs with non-concurrent and only concurrent controls, one sees that the latter allocates more patients to the control group.

### 4.3. Case 3: Given sample sizes in periods 1 and 2

We now assume that $r_{1}>0$ and $r_{2}<1-r_{1}$ are fixed (and not optimized) corresponding to a three-period platform trial in which arm 2 enters later and arm 1 finishes before arm 2 does (Figure 1). As for concurrent controls, we distinguish three scenarios.

(i) If, $r_{1}\geq 1/2$ , as in Case 2, the optimal design allocates all patients $j>N_{1}$ equally to treatment 2 and control, that is, for $p_{0,1}=p_{1,1}=p_{0,2}=p_{2,2}=p_{0,3}=p_{2,3}=1/2$ , and $p_{1,2}=0$ .
(ii) As for concurrent controls, if $r_{1}<1/2$ and in addition $r_{1}+r_{2}<1/2$ , then for all allocations, the maximum variance is the variance of ${\hat{\theta }}_{1}$ . The inclusion of non-concurrent controls can only reduce the variance of ${\hat{\theta }}_{2}$ . Therefore, the optimal design allocates all observations in period 2 to treatment 1 and control.
(iii) If $r_{1}<1/2$ and $r_{1}+r_{2}\geq 1/2$ , we minimize $\max(\mathrm{Var}({\hat{\theta }}_{1}),\mathrm{Var}({\tilde{\theta }}_{2}))$ , by minimizing $\mathrm{Var}({\hat{\theta }}_{1})$ in p for fixed $r_{1}$ and $r_{2}$ . In this case, as in (ii), the two variances are equal under the optimal allocation. As we could not obtain an analytical solution for the optimization problem in this case, we minimized $\mathrm{Var}({\hat{\theta }}_{1})$ in the remaining free variables $p_{1,2},p_{2,2}$ under the constraint of equal variances with the Mathematica function FindMinimum or the r-package nloptr^27^ using the sequential quadratic programing algorithm for nonlinearly constrained gradient-based optimization.^28^ See Section D in the Supplemental Material. Numerical solutions for the optimal allocations in period 2 are shown in dashed lines in Figure 2. As expected, the larger $r_{1}$ , the larger the difference between designs utilizing non-concurrent controls compared to designs with concurrent controls only, as the size of the non-concurrent control group is then larger. The pattern of the optimal allocation rates is similar to the scenario where concurrent controls only are used. However, the ratio of patients assigned to the control group is lower when non-concurrent controls are used. Furthermore, there may be less control patients than patients assigned to arm 1 or 2 (which does not occur if only concurrent controls are used).

## 5. Example and simulation study

We illustrate the optimal allocations in platform trials by means of a phase II placebo-controlled trial in primary hypercholesterolemia.^29^ In this trial, the goal was to evaluate the efficacy of 80 mg of the antibody atorvastatin with SAR236553 as compared to atorvastatin alone. Additionally, there was interest in evaluating other doses and combinations, in particular, to investigate the efficacy of 10 mg of atorvastatin plus SAR236553 compared to atorvastatin alone. The primary endpoint was the percent change in calculated LDL cholesterol from baseline. Patients were randomly assigned according to a 1:1:1 allocation to receive a 80 mg of atorvastatin plus SAR236553, 10 mg of atorvastatin plus SAR236553, or 80 mg of atorvastatin plus placebo.

We revisit the design of this trial to discuss three allocation strategies: equal allocation (1:…:1), square root of k (1:…: $\sqrt{k}$ ) and the proposed optimal allocations.

We assume the total sample size as in the original trial, N=92 , and mean responses and variances in the control group as actually observed in the trial (i.e. a mean of 4.94 with variance 1 in the control), and consider means equal to 5.66 and variance 1 for the experimental treatment arms, which lead to 80% power at 0.025 significance level in a multi-arm design setting using the square root of k allocation. Furthermore, we considered three designs depending on the entry and end of arm 2 in the trial, that is,

- Design with one period only (i.e. multi-arm design), and thus with sample sizes per period $N_{1}=N$ and $N_{2}=N_{3}=0$ as Case 1 (in Section 3.1).
- Design with two periods (arm 2 starts later, but arms 1 and 2 finish at the same time), as Case 2 (in Section 3.2), assuming a sample size of $N_{1}=N/4$ in period 1 and $N_{2}=3N/4$ in period 2.
- Design with three periods (arm 2 starts later and finishes after arm 1 does), as Case 3 (Section 3.3), considering two situations: $N_{1}=N_{2}=N_{3}=N/3$ , and $N_{1}=N/3$ , $N_{2}=4N/9$ and $N_{3}=2N/9$ .

After rounding, we obtain, for example, for the three-period design, $N_{1}=N_{3}=31$ , and $N_{2}=30$ . We consider the analysis for comparing groups based on concurrent data only. For each design configuration and allocation strategy, we describe the sample size per period and arm. Furthermore, we compare the statistical individual power for each treatment control comparison and also evaluate the variance of the estimates by simulating 100,000 trials. In the main manuscript, we report simulation results where no time trends were assumed. In the Supplemental Material (Section B.3), results for trials with time trends are presented.

We start with the standard multi-arm design (Case 1). In this case, as discussed before, the optimal allocation coincides with the square root of k rule (see Table 1 in the Supplemental Material). In such a design, the power of the treatment-control comparisons of treatment 1 is the same as that of treatment 2. When comparing the power of the design with optimal allocations against the design with 1:1 allocation, we can see that there is an increase in power when using the optimal design and the confidence intervals (CIs) are narrower (see Table 2). However, it could also be noted that in this example, the difference is small.

**Table 2.** Power for each design according to the allocation strategy.

				Power	Power	CI width	CI width
Design	$r_{1}$	$r_{2}$	Allocation	$A_{1}$	$A_{2}$	$A_{1}$	$A_{2}$
1-period	1.000	0.000	one	0.798	0.798	1.106	1.106
	1.000	0.000	opt (=sqrt)	0.805	0.805	1.100	1.100
2-period	0.250	0.750	one	0.844	0.671	1.006	1.006
	0.250	0.750	opt	0.772	0.757	0.998	0.998
	0.250	0.750	sqrt	0.851	0.681	0.997	0.997
3-period	0.337	0.326	one	0.720	0.722	1.026	1.151
	0.337	0.326	opt (=sqrt)	0.725	0.724	1.085	1.096
3-period	0.337	0.446	one	0.782	0.687	0.947	1.173
	0.337	0.446	opt	0.737	0.730	1.038	1.055
	0.337	0.446	sqrt	0.784	0.682	0.939	1.157

Here $r_{1}$ and $r_{2}$ are the proportion of patients allocated to periods 1 and 2, respectively; “one” denotes one-to-one allocation, “opt” denotes optimal allocation and “sqrt” denotes the square root of k allocation; “Power A_*i*_” is the estimated power when testing A_*i*_ against control ( i=1,2 ), and “CI Width A_*i*_” refers to the width of the confidence interval for the treatment effect of arm A_*i*_ compared to control. Note that the optimal allocation coincides with the square root of k in the 1-period design and 3-period design with $N_{1}=N_{2}=N_{3}=N/3$ .

Suppose now that the second arm enters while the trial is ongoing, but that it is assumed to end with the first arm. This situation would lead to a two-period design. Table 2 in the Supplemental Material shows the sample sizes per arm and period. We can observe that the designs using optimal allocations and using the root of the k rule differ. The optimal strategy allocates fewer patients to the first arm in the second period, and more to the second arm in the second period, while maintaining an equal allocation between arm 1 and control in the first period as is the case for the one-to-one and square root of k allocations. When comparing power, the results are as expected: the power of testing the efficacy of arm 1 versus control is larger than the power of the comparison regarding arm 2 when using 1:1 allocation and square root of k allocation (see Table 2). Under the optimal allocation strategy, the power and standard errors of effect estimates for both treatment control comparisons are the same. As we minimize maximum variance, we see that the maximum power is smaller under the optimal allocation. Similarly, the (maximum) width of the CIs for the optimal design is smaller than the maximum width of the CI of the other designs.

Finally, consider a design in which arm 2 enters after arm 1, and in addition, the timing when arm 1 ends is fixed at some point before the end of the trial. For the resulting three-period trial, we consider two scenarios. In the first, the total sample sizes for periods 1 and 3 are assumed to be equal. Note that this also implies that the total sample sizes for arms 1 and 2 are equal. Then, in the second period, the optimal allocation is the square root of k allocation. When increasing the sample size of period 2 (such that $r_{3}<r_{1}$ ) this is no longer the case. Moreover, as the period where the control arm is shared increases, with the optimal design also the power increases for larger $r_{2}$ (see Table 2). Under both choices of $r_{2}$ , the power of the arm that achieves the lowest power under the optimal design is larger than the lowest power using the 1:1 and square root of k allocation strategies. See Tables 3 and 4 for the sample size per arm and period assumed for each allocation strategy.

To summarize, for Design 1, the multi-armed trial, as well in the symmetric design where $r_{1}=r_{3}=0.337$ the optimal $\sqrt{2}:1:1$ gives only a small improvement in minimum power (across treatments) compared to equal allocation. However, in the 2-period and non-symmetric 3-period design, the minimum power of the optimized design is substantially larger than for uniform or $\sqrt{2}:1:1$ allocation. This is mainly due to the fact that the latter leads to different variances of the treatment effect estimates (and therefore also different power values) for the two treatment arms.

We also evaluated the type 1 error under the different allocation strategies. In all the considered designs, the type 1 error is controlled (see Table 5 in the Supplemental Material), even if there are time trends (see Section B.3 in the Supplemental Material). However, in the latter case, the variances might slightly deviate because the time trends may increase the variability in the treatment groups.

**Table 3.** Sample size distribution per arm and period according to the allocation strategy for a three-period design where arm 2 starts later and finishes after arm 1 does, and with $N_{1}=N_{2}=N_{3}=N/3$ .

(a) One-to-one					(b) $\sqrt{k}$ allocation					(c) Optimal allocations			
	Period 1	Period 2	Period 3			Period 1	Period 2	Period 3			Period 1	Period 2	Period 3
Arm 2	0	10	16		Arm 2	0	9	16		Arm 2	0	9	16
Arm 1	16	10	0		Arm 1	16	9	0		Arm 1	16	9	0
Control	16	10	16		Control	16	12	16		Control	16	12	16

**Table 4.** Sample size distribution per arm and period according to the allocation strategy for a three-period design where arm 2 starts later and finishes after arm 1 does, and with $N_{1}=N/3$ , $N_{2}=2(N-N_{1})/3$ , and $N_{3}=(N-N_{1})/3$ .

(a) One-to-one					(b) $\sqrt{k}$ allocation					(c) Optimal allocations			
	Period 1	Period 2	Period 3			Period 1	Period 2	Period 3			Period 1	Period 2	Period 3
Arm 2	0	14	10		Arm 2	0	12	10		Arm 2	0	16	10
Arm 1	16	14	0		Arm 1	16	12	0		Arm 1	16	8	0
Control	16	14	10		Control	16	17	10		Control	16	17	10

## 6. Optimal allocation under unequal variances and when minimizing the sum of variances

In the previous sections, we assumed that the responses were distributed with equal variance $\sigma^{2}$ across arms and optimized the allocation rates to minimize the maximum of the variances of the stratified effect estimators. Below, we discuss optimal allocations under unequal variances (Section 6.1) and optimal allocations when minimizing the sum of the variances (Section 6.2). In both sections, we consider a design using concurrent data only and where the entering time of arm 2 and exit time of arm 1 are given by design, that is to say, under Case 3 (see Section 3).

### 6.1. Optimal solutions under unequal variances across arms

Consider the stratified estimators per period in (1) and weights in (2), where the observation of patient j on treatment arm $i_{j}$ ( j=1,…,N , $i_{j}=0,1,2$ ), distributed as $y_{j}∼N(\mu_{i},\sigma_{i}^{2})$ and $\sigma_{i,s}^{2}=(\sigma_{i}^{2}/n_{i,s}+\sigma_{0}^{2}/n_{0,s})$ denotes the variances of the period-wise treatment effect estimates. Then, in analogy to formula (3), the variance of the stratified effect estimator is given by

(15) $$\mathrm{Var}({\hat{\theta }}_{i})=\sum_{s=i,i+1}w_{i,s}^{2}\sigma_{i,s}^{2}=\frac{\sigma_{0}^{2}}{N}\cdot {\left(r_{i}\frac{p_{i,i}p_{0,i}}{p_{i,i}+\frac{\sigma_{i}^{2}}{\sigma_{0}^{2}}\;p_{0,i}}+r_{i+1}\frac{p_{i,i+1}p_{0,i+1}}{p_{i,i+1}+\frac{\sigma_{i}^{2}}{\sigma_{0}^{2}}\;p_{0,i+1}}\right)}^{-1}$$

As above, we aim to find the allocation rates that minimize $\max(\mathrm{Var}({\hat{\theta }}_{1}),\mathrm{Var}({\hat{\theta }}_{2}))$ . The objective function depends on the ratios $\frac{\sigma_{i}^{2}}{\sigma_{0}^{2}},i=1,2$ .

Through numerical optimization, we found that the optimal solution gives rise to the Neyman allocation in periods 1 and 3 (see Supplemental Material D). The optimal solution also varies in period 2 compared to the case of equal variances across arms. Figure 1 in the Supplemental Material illustrates the optimal allocation rates in a design with equal versus unequal variances across arms. The pattern is similar to the optimal solutions with equal variances (in Figure 2) with respect to the dependence on $r_{1}$ and $r_{2}$ : as $r_{2}$ increases, the allocation ratio for arm 2 also increases, while the allocation ratio for arm 1 decreases. Similarly, for $r_{1}$ , the allocation ratios increase for arm 1 and decrease for arm 2 as $r_{1}$ increases. Moreover, compared to the case of equal variances, more subjects are allocated to the arm with greater variability.

### 6.2. Optimal solutions when minimizing the sum of variances

As in Section 6.1, we consider a design using concurrent data, the stratified estimators per period in (1) and weights in (2). Instead of minimizing the maximum of the variances of the stratified effect estimators, we now use the sum of the variances as the objective function to be minimized. Thus, given a fixed overall sample size N , we aim to find the allocation probabilities p that minimizes $\mathrm{Var}({\hat{\theta }}_{1})+\mathrm{Var}({\hat{\theta }}_{2})$ . The optimal designs can be derived by numerical optimization (see Supplemental Material D).

As in the optimization using the maximum of the variances as the objective function, the optimal design leads to equal allocation between the active arm and the control in periods 1 and 3. Furthermore, in period 2, both optimal designs coincide in the optimal allocation rates when $r_{1}=r_{3}$ where the solution gives equal allocation between the treatment arms and square root of k=2 allocation for the control. For the rest of the scenarios where $r_{1}\neq r_{3}$ , the optimal solutions do not coincide (see Figure 2 in the Supplemental Material). In fact, for $r_{1}<r_{2}$ , when minimizing the sum of the variances, fewer subjects are allocated to arm 1 and more to arm 2 compared to the optimal solutions minimizing the maximum of the variances, while the opposite happens for $r_{1}>r_{2}$ . In addition, more subjects are allocated to the control when minimizing the sum of variances instead of the maximum.

## 7. Discussion

We derived optimal allocation rules for platform trials, minimizing the maximum variance of the treatment effect estimators of treatment-control comparisons. The most efficient design is the multi-arm trial, where all treatments start and end at the same time. However, this may not be feasible if, for example, not all treatments are available at the start. Under the assumption that some treatments enter the trial at a later time point, we showed that in the optimal design, all treatments finish at the end of the platform trial. This again, however, will in general not be practical. Therefore, we considered optimal designs under constraints on the entry and exit times of treatment arms.

In contrast to earlier work, we performed the optimization assuming that the analyses to compare the efficacy of treatments against control are adjusted with the categorical factor time period. These periods are defined by the time intervals in which no arm enters or leaves the study. Adjusted analyses are recommended in this case to avoid potential biases caused by time trends. Note that the optimal allocation depends on the chosen analysis model.

As an objective function, we considered the maximum of the variances of the two treatment effect estimators (6). As a consequence, under optimal allocation the variances of the treatment effect estimates are either equal, or as similar a possible, given the design restrictions with regard to the size of the periods. If the treatment effects are equal, this also results in an equal power for each of the arms. This is a desirable property, especially in multi-sponsor platform trials, as it gives equal chances to the different treatments. In Section 6, we considered the sum of the variances of the treatment effect estimates as an alternative objective function. The resulting optimal treatment allocation is the same in symmetric settings, where $r_{1}=r_{3}$ . In all other settings, the proportion of patients allocated to the control group is larger compared to the optimal allocation for the objective function (6). Other objective functions that have been considered are the probability to reject at least one null hypothesis or the sum (or average) of the individual rejection probabilities.

In this work, we assumed that the total sample size of the platform trial is known. Although the total sample size is only a scaling factor in the optimization problem, this assumption might be unrealistic. However, an equivalent strategy to the one followed to optimize the allocation rates would be to consider a targeted minimum precision (corresponding to the maximum variance) for the estimates of the treatment effects, assuming that these are equal, and minimize the total sample size of the trial. To this end, we need to search for the total sample sizes N such that the minimum precision of the corresponding optimal design is equal to the target precision. As the optimized minimum precision is monotonic in N , this corresponds to a simple numerical root finding.

Changing the allocation ratio in a clinical trial has been controversially discussed in the context of response adaptive randomization.^30–33^ The main concern is the need to adjust for potential time trends, which leads to statistical inefficiency. Also, for platform trials, we saw that the statistically most efficient design is a multi-arm trial, where allocation ratios stay constant over time. However, if it is not feasible to start and complete all arms at the same time, but some arms enter the trial at a later time point, or complete recruitment before the end of the platform trial, a change in the allocation ratios is optimal, also for analysis procedures that adjust for the time trends.

The testing approach considered in this article, which adjusts for time trends by including a period effect, is in line with the recent Food and Drug Administration (FDA) draft guidance on Master Protocols^34^ which suggests the use of stratified analyses to avoid bias caused by time trends if the allocation ratios are modified during the trial. Our findings also support the recommendations regarding the choice of allocation ratios: also in the draft guidance, a randomization process that assigns more subjects to the control arm than each individual treatment arm is recommended. This can increase the power for treatment-control comparisons for a given total sample size.

In this article, we optimized the allocation ratios under the assumption that the times when the second treatment enters or leaves the platform are known. In many realistic settings, these times may, however, be unknown at the start of the trial. In the resulting, optimal trial designs, however, the optimal allocation ratio in the first period is always 1:1 randomization and, thus, does not depend on $r_{1}$ and $r_{2}$ . The optimal allocation ratios for period 2 can be computed when treatment 2 enters the platform and $r_{2}$ determining when treatment 1 should be completed is fixed. This holds for both, analysis including non-concurrent controls and concurrent controls only. If it is desirable to use equal allocation among the experimental treatment arms, one can choose $r_{2}$ such that the optimal design satisfies this condition (see Figure 2). For the analysis using concurrent controls only, the optimal allocation in period 2 is then a $1:1:\sqrt{2}$ allocation.

Our article aims to advance the understanding of optimal allocation principles in platform trials. To accomplish this, we centered our focus on a trial design with two experimental arms and a shared control arm without interim analyses. The inclusion of interim analyses has two consequences. First, the analysis model needs to be modified, as the size of the periods can depend on the interim results. Second, optimization under the assumption of a fixed overall sample size is no longer applicable, as, due to early stopping, the overall sample size depends on the trial outcomes. Instead one could, for example optimize the allocation ratios fixing the overall expected sample size, averaged over a prior on the effect sizes. Even without interim analyses, optimizing the allocation ratios in platform trials with more than two experimental treatment arms becomes more complex, due to the larger number of parameters. In addition, in larger platform trials a feasible optimal allocation rule must not depend on the sample sizes in all future periods, because they may be unknown in advance (as they depend on the entry times of future treatment arms). A simplified strategy could be to set the allocation ratios between treatment arms to 1 (equal allocation) and to optimize the allocation rate to the control arm only. The exit time of the treatment arms could be specified such that the variance of the respective treatment effect estimate reaches a certain threshold. The derivation of optimal allocation rules for platform trials with more than two arms and interim analyses is an open problem and subject to further research.

## Supplemental Material

sj-pdf-1-smm-10.1177_09622802241239008 - Supplemental material for Optimal allocation strategies in platform trials with continuous endpoints Supplemental material, sj-pdf-1-smm-10.1177_09622802241239008 for Optimal allocation strategies in platform trials with continuous endpoints by Marta Bofill Roig, Ekkehard Glimm, Tobias Mielke and Martin Posch in Statistical Methods in Medical Research

## Appendix

For trials using non-concurrent controls and assuming a fixed sample size in period 1 (Case 2 in Section 4.2), the formulas for the optimal allocation ratios in period 2 are

$$\begin{array}{rl} p_{22} & =1+\frac{1}{4\sqrt{3}\sqrt{a(1-r_{1})}}\left(\sqrt{-b}-\sqrt{4+8a+a^{2}-12(2+a)r_{1}+12r_{1}\sqrt{3}\sqrt{\frac{a^{3}(1-r_{1})}{-b}}+9r_{1}^{2}}\right)\\ p_{12} & =\left(1-\sqrt{4r_{1}p_{22}/(1-r_{1})-r_{1}+4p_{22}^{2}/(1-r_{1}{)}^{2}-4p_{22}/(1-r_{1})+1}-r_{1}\right)/(2(1-r_{1})) \end{array}$$

where $a=\sqrt[{3}]{9r_{1}(3(r_{1}-4)r_{1}+4)+6\sqrt{3}\sqrt{r_{1}(16-9r_{1}(3(r_{1}-4)r_{1}+8))}+8}$ and $b=6(a-4)r_{1}+(a-2{)}^{2}+9r_{1}^{2}$ .
