# Supplementary material for: Optimal allocation strategies in platform trials with continuous endpoints
Source: Stat Methods Med Res. 2024 Mar 20;33(5):858–74. doi: 10.1177/09622802241239008 (PMC11041082; doi:10.1177/09622802241239008)
Supplement: sj-pdf-1-smm-10.1177_09622802241239008 - Supplemental material for Optimal allocation strategies in platform trials with continuous endpoints [file sj-pdf-1-smm-10.1177_09622802241239008.pdf]

Supplementary material of "Optimal allocation strategies in platform trials with continuous endpoints"

Marta Bofill Roig[1], Ekkehard Glimm[2], Tobias Mielke[3], and Martin Posch*[1]

[1]Section for Medical Statistics, Center for Medical Data Science, Medical University of Vienna, Vienna
[2]Advanced Methodology and Data Science, Novartis Pharma AG, Basel
[3]Statistics and Decision Sciences, Janssen-Cilag GmbH

Contents

A Variance derivations **2**

A.1 Variance for effect estimators using concurrent controls only 2

A.2 Variance for the effect estimator using concurrent and non-concurrent controls 2

A.2.1 Effect estimator using concurrent and non-concurrent controls 3

A.2.2 Two-period trial 4

A.2.3 Three-period trial 5

B Additional simulation results **5**

B.1 Sample sizes per period and arm 5

B.2 Results under the null hypothesis 6

B.3 Results for trials with time trends 6

C Additional optimisation results **9**

C.1 Optimal solutions under unequal variances across arms 9

C.2 Optimal solutions when minimising the sum of variances 10

D Mathematica files **11**

*martin.posch@meduniwien.ac.at

In this supplementary material, we provide the calculations of the variance estimators (Section A) and additional results concerning the simulation study (Section B). Mathematica and R code to reproduce the results are available at `https://github.com/MartaBofillRoig/Allocation`. However, for the reader's convenience, we also include the Mathematica files at the end of this document.

A Variance derivations

Consider the same notation used in Section 2 of the article and let $n_{i,s}, i = 0, 1, 2, s = 1, 2, 3$ be the sample sizes in arm i, period s of the platform trial design.

A.1 Variance for effect estimators using concurrent controls only

Consider the period-wise treatment effect estimators of the form

$$\widehat{\theta}_i = \sum_{s=i,i+1} w_{i,s} \cdot \hat{\theta}_{i,s}$$

where $\hat{\theta}_{i,s} = \bar{y}_{i,s} - \bar{y}_{0,s}$ is the treatment effect estimate in period s for arm i ($i = 1, 2$), where $\bar{y}_{i,s}$ and $\bar{y}_{0,s}$ are the sample means in period s for experimental and control arms, and the weights are given by

$$w_{1,s} = \frac{\frac{1}{\sigma^2_{1,s}}}{\frac{1}{\sigma^2_{1,1}} + \frac{1}{\sigma^2_{1,2}}} \quad \text{and} \quad w_{2,s} = \frac{\frac{1}{\sigma^2_{2,s}}}{\frac{1}{\sigma^2_{2,2}} + \frac{1}{\sigma^2_{2,3}}}, \tag{1.a}$$

where $\sigma^2_{i,s} = \text{Var}(\hat{\theta}_{i,s})$ denotes the variances of the estimates per period, and is given by $\sigma^2_{i,s} = \sigma^2(1/n_{i,s} + 1/n_{0,s})$.

Then, the variance of the estimator is then given by:

$$\begin{aligned}
\text{Var}(\widehat{\theta}_i) &= \sum_{s=i,i+1} w^2_{i,s}\sigma^2_{i,s} = \sum_{s=i,i+1} \left(\frac{\frac{1}{\sigma^2_{i,s}}}{\frac{1}{\sigma^2_{i,i}} + \frac{1}{\sigma^2_{i,i+1}}}\right)^2 \sigma^2_{i,s} = \sum_{s=i,i+1} \frac{\frac{1}{\sigma^2_{i,s}}}{\left(\frac{1}{\sigma^2_{i,i}} + \frac{1}{\sigma^2_{i,i+1}}\right)^2} = \frac{1}{\frac{1}{\sigma^2_{i,i}} + \frac{1}{\sigma^2_{i,i+1}}} \\
&= \sigma^2 \cdot \left(\frac{1}{\frac{1}{n_{i,i}} + \frac{1}{n_{0,i}}} + \frac{1}{\frac{1}{n_{i,i+1}} + \frac{1}{n_{0,i+1}}}\right)^{-1} = \sigma^2 \cdot \left(\frac{n_{i,i}n_{0,i}}{n_{i,i} + n_{0,i}} + \frac{n_{i,i+1}n_{0,i+1}}{n_{i,i+1} + n_{0,i+1}}\right)^{-1} \\
&= \frac{\sigma^2}{N} \cdot \left(r_i \frac{p_{i,i}p_{0,i}}{p_{i,i} + p_{0,i}} + r_{i+1} \frac{p_{i,i+1}p_{0,i+1}}{p_{i,i+1} + p_{0,i+1}}\right)^{-1}.
\end{aligned}$$

as $p_{i,s} = \frac{n_{i,s}}{r_s N_s}$ and $r_s = \frac{N_s}{N}$.

A.2 Variance for the effect estimator using concurrent and non-concurrent controls

In this section, we first derive the expression of the effect estimators using concurrent and non-concurrent controls and then give the variance expressions. Note that in what follows, the treatment effect estimator for arm 1 uses concurrent and non-concurrent controls as well, as arm 2 does. However, in the case of arm 1, non-concurrent controls are those patients allocated to the control in period 3. For the purpose of optimal allocations in this paper, we are only interested in the estimator of arm 2, but we give here the expressions of the estimator of arm 1 for completeness.

In Section A.2.1, we derive the expressions of the estimators and the corresponding variances when utilising non-concurrent controls together with concurrent controls. In Sections A.2.2 and A.2.3, we give the expressions for the particular case of a platform trial with two-periods and three-periods, respectively. The expressions given in the latter sections are the ones we use in Section 4 of the manuscript.

A.2.1 Effect estimator using concurrent and non-concurrent controls

We assume a linear model $E(\mathbf{y}) = \boldsymbol{X\theta}$ where $\boldsymbol{\theta} = (\mu_1, \mu_2, \mu_3, \theta_1, \theta_2)'$ is the parameter vector with baseline stagewise responses μ_s and treatment effects θ_i. $\boldsymbol{X}$ is the design matrix and $\mathbf{y}$ the vector of independent observations with a constant variance σ^2 (without loss of generality, $\sigma = 1$).

The Gauss-Markov (best linear unbiased) estimator of $\boldsymbol{\theta}$ is $\hat{\boldsymbol{\theta}} = (\boldsymbol{X'X})^{-1}\boldsymbol{X'}\mathbf{y}$. It is best linear unbiased in the sense that for any other estimate $\tilde{\boldsymbol{\theta}} = \boldsymbol{Ay}$ with $E(\tilde{\boldsymbol{\theta}}) = \boldsymbol{\theta}$, $Cov(\tilde{\boldsymbol{\theta}}) - Cov(\hat{\boldsymbol{\theta}})$ is positive semidefinite which in turn implies that the variance of any component of $\hat{\boldsymbol{\theta}}$ is at a minimum among these unbiased linear estimates. We have $Cov(\hat{\boldsymbol{\theta}}) = \sigma^2 (\boldsymbol{X'X})^{-1}$.

In the specific case here, the design matrix is

$$\boldsymbol{X} = \begin{pmatrix} \mathbf{1}_{n_{0,1}} & \mathbf{0} & \mathbf{0} & \mathbf{0} & \mathbf{0} \\ \mathbf{1}_{n_{1,1}} & \mathbf{0} & \mathbf{0} & \mathbf{1}_{n_{1,1}} & \mathbf{0} \\ \mathbf{0} & \mathbf{1}_{n_{0,2}} & \mathbf{0} & \mathbf{0} & \mathbf{0} \\ \mathbf{0} & \mathbf{1}_{n_{1,2}} & \mathbf{0} & \mathbf{1}_{n_{1,2}} & \mathbf{0} \\ \mathbf{0} & \mathbf{1}_{n_{2,2}} & \mathbf{0} & \mathbf{0} & \mathbf{1}_{n_{2,2}} \\ \mathbf{0} & \mathbf{0} & \mathbf{1}_{n_{0,3}} & \mathbf{0} & \mathbf{0} \\ \mathbf{0} & \mathbf{0} & \mathbf{1}_{n_{2,3}} & \mathbf{0} & \mathbf{1}_{n_{2,3}} \end{pmatrix}$$

where $\mathbf{1}_x$ denotes a vector of x ones. Hence,

$$\boldsymbol{X'X} = \begin{pmatrix} N_1 & 0 & 0 & n_{1,1} & 0 \\ 0 & N_2 & 0 & n_{1,2} & n_{2,2} \\ 0 & 0 & N_3 & 0 & n_{2,3} \\ n_{11} & n_{1,2} & 0 & n_{1,1} + n_{1,2} & 0 \\ 0 & n_{2,2} & n_{2,3} & 0 & n_{2,2} + n_{2,3} \end{pmatrix}$$

where N_s is the sample size per period, that is, $N_1 = n_{0,1} + n_{1,1}$, $N_2 = n_{0,2} + n_{1,2} + n_{2,2}$, $N_3 = n_{0,3} + n_{2,3}$. We can use the technique of inverting block-partitioned matrices here. If we write

$$\boldsymbol{A} = \begin{pmatrix} N_1 & 0 & 0 \\ 0 & N_2 & 0 \\ 0 & 0 & N_3 \end{pmatrix},$$

$$\boldsymbol{C} = \begin{pmatrix} n_{1,1} + n_{1,2} & 0 \\ 0 & n_{2,2} + n_{2,3} \end{pmatrix},$$

and

$$\boldsymbol{B} = \begin{pmatrix} n_{1,1} & 0 \\ n_{1,2} & n_{2,2} \\ 0 & n_{2,3} \end{pmatrix},$$

then, by this technique, we obtain

$$cov(\widetilde{\theta}_1, \widetilde{\theta}_2) = \left(\boldsymbol{C} - \boldsymbol{B'A^{-1}B}\right)^{-1} = f^{-1} \cdot \begin{pmatrix} n_{2,2} + n_{2,3} - \frac{n_{2,2}^2}{N_2} - \frac{n_{2,3}^2}{N_3} & \frac{n_{1,2}n_{2,2}}{N_2} \\ \frac{n_{1,2}n_{2,2}}{N_2} & n_{1,1} + n_{1,2} - \frac{n_{1,1}^2}{N_1} - \frac{n_{1,2}^2}{N_2} \end{pmatrix} \tag{1.b}$$

where $f = \left(n_{1,1} + n_{1,2} - \frac{n_{1,1}^2}{N_1} - \frac{n_{1,2}^2}{N_2}\right) \cdot \left(n_{2,2} + n_{2,3} - \frac{n_{2,2}^2}{N_2} - \frac{n_{2,3}^2}{N_3}\right) - \left(\frac{n_{1,2}n_{2,2}}{N_2}\right)^2$.

Regarding the point estimates, it is possible to simplify to

$$\begin{pmatrix} \widetilde{\theta}_1 \\ \widetilde{\theta}_2 \end{pmatrix} = \left(\boldsymbol{C} - \boldsymbol{B'A^{-1}B}\right)^{-1} \left(-\boldsymbol{B'A^{-1}} \vdots\, \boldsymbol{I}_2\right) \begin{pmatrix} N_1\bar{y}_{.,1} \\ N_2\bar{y}_{.,2} \\ N_3\bar{y}_{.,3} \\ n_{1,1}\bar{y}_{1,1} + n_{1,2}\bar{y}_{1,2} \\ n_{2,2}\bar{y}_{2,2} + n_{1,2}\bar{y}_{2,3} \end{pmatrix} = \tag{1.c}$$

$$\left(\boldsymbol{C} - \boldsymbol{B'A^{-1}B}\right)^{-1} \begin{pmatrix} n_{1,1}(\bar{y}_{1,1} - \bar{y}_{.,1}) + n_{1,2}(\bar{y}_{1,2} - \bar{y}_{.,2}) \\ n_{2,2}(\bar{y}_{2,2} - \bar{y}_{.,2}) + n_{2,3}(\bar{y}_{2,3} - \bar{y}_{.,3}) \end{pmatrix}$$

where $\bar{y}_{i,s}$ is the pooled mean per arm and period, and $\bar{y}_{\cdot,s}$ is the pooled mean per period. Note, however, that $\widetilde{\theta}_i$ cannot, in general, be represented as a weighted average of stagewise treatment effect contrasts, i.e. as $\sum_{i=1}^{2}\sum_{s=1}^{3} w_{i,s}\cdot(\bar{y}_{i,s}-\bar{y}_{0,s})$.

We focus on the treatment effect estimator for treatment 2 only, given that this is the arm in which we use non-concurrent controls. Assume now that $N_s = r_s N$ is the sample size in period s and $r_{i,s} = \frac{n_{i,s}}{N}$ is the fraction of patients in arm $i = 0,1,2$ and period s among all patients such that $r_s = \sum_{i\in I_s} r_{i,s}$ and $r_1+r_2+r_3 = 1$. $\mathrm{Var}(\widetilde{\theta}_1)$ and $\mathrm{Var}(\widetilde{\theta}_2)$ are the diagonal of (1.b). With this notation, we can write the variance as

$$\mathrm{Var}(\widetilde{\theta}_2) = \frac{r_1 p_{1,1}(1-p_{1,1}) + r_2 p_{1,2}(1-p_{1,2})}{(r_1 p_{1,1}(1-p_{1,1}) + r_2 p_{1,2}(1-p_{1,2}))(r_3 p_{2,3}(1-p_{2,3}) + r_2 p_{2,2}(1-p_{2,2})) - r_2^2 p_{1,2}^2 p_{2,2}^2}\cdot\frac{1}{N} \quad (1.d)$$

where $p_{i,s} = \frac{r_{i,s}}{r_s}$ is the proportion of patients in treatment group i of period s.

We assume that r_1 is fixed. Minimizing $\mathrm{Var}(\widetilde{\theta}_i)$ is equivalent to maximizing $\mathrm{Var}(\widetilde{\theta}_i)^{-1}$. Denoting by $q_{js} = p_{js}(1-p_{js})$, we obtain

$$\mathrm{Var}(\widetilde{\theta}_2)^{-1} \propto r_3 q_{23} + r_2 q_{22} - \frac{r_2^2 p_{12}^2 p_{22}^2}{r_1 q_{11} + r_2 q_{12}}. \quad (1.e)$$

A.2.2 Two-period trial

Consider a two-period trial, where arm 2 enters later and finishes at the same time that arm 1, as illustrated in Figure 3 of the paper. The treatment effect estimator for treatment 2 using non-concurrent controls can then be written as

$$\widetilde{\theta}_2 = \hat{\theta}_{2,2} + \rho(\hat{\theta}_{1,1} - \hat{\theta}_{1,2})$$

where

$$\rho = \frac{\frac{1}{n_{0,2}}}{\frac{1}{n_{0,1}} + \frac{1}{n_{0,2}} + \frac{1}{n_{1,1}} + \frac{1}{n_{1,2}}}$$

The variance of the estimator $\widetilde{\theta}_2$ is then

$$\begin{aligned}
\mathrm{Var}(\tilde{\theta}_2) &= \mathrm{Var}(\hat{\theta}_{2,2}) + \rho^2\mathrm{Var}(\hat{\theta}_{1,1}) + \rho^2\mathrm{Var}(\hat{\theta}_{1,2}) - 2\rho\mathrm{Cov}(\hat{\theta}_{2,2},\hat{\theta}_{1,2}) = \sigma_{2,2}^2 + \rho^2(\sigma_{1,1}^2+\sigma_{1,2}^2) - 2\rho\frac{\sigma^2}{n_{0,2}} \\
&= \sigma^2\left(\frac{1}{n_{2,2}} + \frac{1}{n_{0,2}}\right) - 2\rho\sigma^2\frac{1}{n_{0,2}} + \rho^2\sigma^2\left(\frac{1}{n_{1,1}} + \frac{1}{n_{0,1}} + \frac{1}{n_{1,2}} + \frac{1}{n_{0,2}}\right) \\
&= \sigma^2\left(\frac{1}{n_{2,2}} + \frac{1}{n_{0,2}}\right) - 2\sigma^2\cdot\frac{\left(\frac{1}{n_{0,2}}\right)^2}{\frac{1}{n_{0,1}} + \frac{1}{n_{0,2}} + \frac{1}{n_{1,1}} + \frac{1}{n_{1,2}}} + \sigma^2\cdot\frac{\left(\frac{1}{n_{0,2}}\right)^2}{\frac{1}{n_{0,1}} + \frac{1}{n_{0,2}} + \frac{1}{n_{1,1}} + \frac{1}{n_{1,2}}} \\
&= \sigma^2\left(\frac{1}{n_{2,2}} + \frac{1}{n_{0,2}} - \frac{\left(\frac{1}{n_{0,2}}\right)^2}{\frac{1}{n_{0,1}} + \frac{1}{n_{0,2}} + \frac{1}{n_{1,1}} + \frac{1}{n_{1,2}}}\right)
\end{aligned}$$

By taking into account $p_{i,s} = \frac{n_{i,s}}{r_s N_s}$, then:

$$\mathrm{Var}(\tilde{\theta}_2) = \frac{\sigma^2}{N}\left(r_2 q_{2,2} - \frac{r_2^2 p_{1,2}^2 p_{2,2}^2}{r_1 q_{1,1} + r_2 q_{1,2}}\right)^{-1} \quad (1.f)$$

A.2.3 Three-period trial

Suppose now a three-period platform trial in which arm 2 enters later and arm 1 finishes before arm 2 does (see Figure 1 in the paper for an illustration). The treatment effect estimator for comparing arm 2 against control when using the model-based approaches is

$$\widetilde{\theta}_2 = \omega_{1,1}(\hat{y}_{1,1} - \hat{y}_{\cdot,1}) + \omega_{1,2}(\hat{y}_{1,2} - \hat{y}_{\cdot,2}) + \omega_{2,2}(\hat{y}_{2,2} - \hat{y}_{\cdot,2}) + \omega_{2,3}(\hat{y}_{2,3} - \hat{y}_{\cdot,3}) \quad (1.g)$$

where $\hat{y}_{\cdot,s}$ is the pooled mean per period, and where $\omega_{i,s}$ are given by:

$$\begin{aligned}
\omega_{1,1} &= \Big(n_{1,1}(n_{0,1}+n_{1,1})n_{1,2}n_{2,2}(n_{0,3}+n_{2,3})\Big) \cdot \Big(n_{1,1}n_{1,2}(n_{0,2}n_{0,3}n_{2,2}+n_{0,3}n_{2,2}n_{2,3}+n_{0,2}(n_{0,3}+n_{2,2})n_{2,3}) \\
&\quad +n_{0,1}(n_{0,3}n_{1,1}n_{1,2}n_{2,2}+(n_{0,3}n_{1,1}n_{1,2}+n_{1,1}n_{1,2}n_{2,2}+n_{0,3}(n_{1,1}+n_{1,2})n_{2,2})n_{2,3} \\
&\quad +n_{0,2}(n_{1,1}+n_{1,2})(n_{2,2}n_{2,3}+n_{0,3}(n_{2,2}+n_{2,3})))\Big)^{-1} \\
\omega_{1,2} &= \Big((n_{0,1}+n_{1,1})n_{1,2}^2n_{2,2}(n_{0,3}+n_{2,3})\Big) \cdot \Big(n_{1,1}n_{1,2}(n_{0,2}n_{0,3}n_{2,2}+n_{0,3}n_{2,2}n_{2,3}+n_{0,2}(n_{0,3}+n_{2,2})n_{2,3})+ \\
&\quad +n_{0,1}(n_{0,3}n_{1,1}n_{1,2}n_{2,2}+(n_{0,3}n_{1,1}n_{1,2}+n_{1,1}n_{1,2}n_{2,2}+n_{0,3}(n_{1,1}+n_{1,2})n_{2,2})n_{2,3} \\
&\quad +n_{0,2}(n_{1,1}+n_{1,2})(n_{2,2}n_{2,3}+n_{0,3}(n_{2,2}+n_{2,3})))\Big)^{-1} \\
\omega_{2,2} &= \Big(n_{2,2}(n_{1,1}n_{1,2}(n_{0,2}+n_{2,2})+n_{0,1}(n_{1,1}n_{1,2}+n_{0,2}(n_{1,1}+n_{1,2})+(n_{1,1}+n_{1,2})n_{2,2}))(n_{0,3}+n_{2,3})\Big) \cdot \\
&\quad \cdot\Big(n_{1,1}n_{1,2}(n_{0,2}n_{0,3}n_{2,2}+n_{0,3}n_{2,2}n_{2,3}+n_{0,2}(n_{0,3}+n_{2,2})n_{2,3})+ \\
&\quad +n_{0,1}(n_{0,3}n_{1,1}n_{1,2}n_{2,2}+(n_{0,3}n_{1,1}n_{1,2}+n_{1,1}n_{1,2}n_{2,2}+n_{0,3}(n_{1,1}+n_{1,2})n_{2,2})n_{2,3}+ \\
&\quad +n_{0,2}(n_{1,1}+n_{1,2})(n_{2,2}n_{2,3}+n_{0,3}(n_{2,2}+n_{2,3})))\Big)^{-1} \\
\omega_{2,3} &= \Big((n_{1,1}n_{1,2}(n_{0,2}+n_{2,2})+n_{0,1}(n_{1,1}n_{1,2}+n_{0,2}(n_{1,1}+n_{1,2})+(n_{1,1}+n_{1,2})n_{2,2}))n_{2,3}(n_{0,3}+n_{2,3})\Big) \cdot \\
&\quad \cdot\Big(n_{1,1}n_{1,2}(n_{0,2}n_{0,3}n_{2,2}+n_{0,3}n_{2,2}n_{2,3}+n_{0,2}(n_{0,3}+n_{2,2})n_{2,3})+ \\
&\quad +n_{0,1}(n_{0,3}n_{1,1}n_{1,2}n_{2,2}+(n_{0,3}n_{1,1}n_{1,2}+n_{1,1}n_{1,2}n_{2,2}+n_{0,3}(n_{1,1}+n_{1,2})n_{2,2})n_{2,3}+ \\
&\quad +n_{0,2}(n_{1,1}+n_{1,2})(n_{2,2}n_{2,3}+n_{0,3}(n_{2,2}+n_{2,3})))\Big)^{-1}
\end{aligned}$$

The variance of the estimator $\widetilde{\theta}_2$ is given in (1.d).

B Additional simulation results

B.1 Sample sizes per period and arm

In what follows, we describe the sample size distribution per period and arm according to each design configuration and allocation strategy.

The considered designs are:

1. Design with one period only (that is, multi-arm design), and thus with sample sizes per period $N_1 = N$ and $N_2 = N_3 = 0$.
2. Design with two periods (arm 2 starts later, but arms 1 and 2 finish at the same time), assuming a sample size of $N_1 = N/4$ in period 1 and $N_2 = 3N/4$ in period 2.
3. Design with three periods (arm 2 starts later and finishes after arm 1 does), where considering two situations: $N_1 = N_2 = N_3 = N/3$, and $N_1 = N/3$ $N_2 = 2(N - N_1)/3$ and $N_3 = (N - N_1)/3$.

where $N = 92$. The following tables depict the sample size per arm and period according to each design and with respect to the allocation strategy.

Table 1: Sample size distribution per arm and period according to the allocation strategy for a multi-arm trial design (one-period design).

	Period 1	Period 2	Period 3
Arm 2	31	0	0
Arm 1	31	0	0
Control	31	0	0

(a) One-to-one

	Period 1	Period 2	Period 3
Arm 2	27	0	0
Arm 1	27	0	0
Control	38	0	0

(b) $\sqrt{k}$ allocation

	Period 1	Period 2	Period 3
Arm 2	27	0	0
Arm 1	27	0	0
Control	38	0	0

(c) Optimal allocations

Table 2: Sample size distribution per arm and period according to the allocation strategy for a two-period design where arm 2 starts later and finishes when arm 1 does, and with sample size of $N_1 = N/4$ in period 1 and $N_2 = 3N/4$ in period 2.

	Period 1	Period 2	Period 3
Arm 2	0	23	0
Arm 1	12	23	0
Control	12	23	0

(a) One-to-one

	Period 1	Period 2	Period 3
Arm 2	0	20	0
Arm 1	12	20	0
Control	12	29	0

(b) $\sqrt{k}$ allocation

	Period 1	Period 2	Period 3
Arm 2	0	27	0
Arm 1	12	12	0
Control	12	30	0

(c) Optimal allocations

Table 3: Sample size distribution per arm and period according to the allocation strategy for a three-period design where arm 2 starts later and finishes after arm 1 does, and with $N_1 = N_2 = N_3 = N/3$.

	Period 1	Period 2	Period 3
Arm 2	0	10	16
Arm 1	16	10	0
Control	16	10	16

(a) One-to-one

	Period 1	Period 2	Period 3
Arm 2	0	9	16
Arm 1	16	9	0
Control	16	12	16

(b) $\sqrt{k}$ allocation

	Period 1	Period 2	Period 3
Arm 2	0	9	16
Arm 1	16	9	0
Control	16	12	16

(c) Optimal allocations

Table 4: Sample size distribution per arm and period according to the allocation strategy for a three-period design where arm 2 starts later and finishes after arm 1 does, and with $N_1 = N/3$, $N_2 = 2(N - N_1)/3$ and $N_3 = (N - N_1)/3$.

	Period 1	Period 2	Period 3
Arm 2	0	14	10
Arm 1	16	14	0
Control	16	14	10

(a) One-to-one

	Period 1	Period 2	Period 3
Arm 2	0	12	10
Arm 1	16	12	0
Control	16	17	10

(b) $\sqrt{k}$ allocation

	Period 1	Period 2	Period 3
Arm 2	0	16	10
Arm 1	16	8	0
Control	16	17	10

(c) Optimal allocations

B.2 Results under the null hypothesis

In this section, we report the results of the simulations under the null hypothesis. In this case, we considered means for the treatment arms equal to 4.94 as in the control group, and variances equal to 1. Table 5 summarizes the results. We can observe that the type 1 error is controlled in all considered scenarios.

B.3 Results for trials with time trends

In this section, we present the results of the case study for trials with time trends. We considered the designs presented in the previous section, and the same parameter settings as in the paper: a total sample size of $N = 92$, the mean in the control is 4.94 with variance 1, and means equal to 5.66 and variance 1 in the treatment arms. In this simulation, however, we considered step-wise time trends. Specifically, the data are

Table 5: Type 1 error (T1E) rates for each design according to the allocation strategy. Here r_1 and r_2 are the proportion of patients allocated to periods 1 and 2, respectively; "one" denotes one-to-one allocation, "opt" denotes optimal allocation and "sqrt" denotes the square root of k allocation; "T1E A_i" is the estimated type 1 error when testing A_i against control ($i = 1, 2$), and "CI Width A_i" refers to the width of the confidence interval for the treatment effect of arm A_i against control.

Design	r_1	r_2	Allocation	T1E *A1*	T1E *A2*	CI Width *A1*	CI Width *A2*
1-period	1.000	0.000	one	0.025	0.026	1.106	1.106
	1.000	0.000	opt	0.025	0.026	1.099	1.100
	1.000	0.000	sqrt	0.025	0.025	1.100	1.100
2-period	0.250	0.750	one	0.025	0.025	1.006	1.006
	0.250	0.750	opt	0.026	0.025	0.997	0.997
	0.250	0.750	sqrt	0.025	0.025	0.997	0.997
3-period	0.337	0.326	one	0.025	0.025	1.026	1.151
	0.337	0.326	opt	0.025	0.025	1.084	1.095
	0.337	0.326	sqrt	0.025	0.024	1.025	1.149
3-period	0.337	0.446	one	0.025	0.025	0.947	1.173
	0.337	0.446	opt	0.025	0.026	1.038	1.055
	0.337	0.446	sqrt	0.025	0.026	0.938	1.156

generated according to the model

$$E(Y_j) = \eta_0 + \sum_{i=1,2} \theta_i \cdot I(i_j = i) + f(t_j), \tag{2.h}$$

where η_0 is the control response in period 1, θ_k is the treatment effect for arm k, $f(\cdot)$ represents the time trend function and t_j is the calendar time when patient j is enrolled in the trial, where $f(j) = \lambda I(j > N_1)$. Here λ represents the strength of the time trend, which is considered to be $\lambda = 0.25$ in the simulations.

In the following tables, we present the resulting power and type 1 error for each design and allocation strategy. We can see that the results also hold for trials with time trends and that the type 1 error is maintained.

Table 6: Power for each design according to the allocation strategy for a trial with stepwise time trends (see (2.h) for the data generation). Here r_1 and r_2 are the proportion of patients allocated to periods 1 and 2, respectively; "one" denotes one-to-one allocation, "opt" denotes optimal allocation and "sqrt" denotes the square root of k allocation; "Power A_i" is the estimated type 1 error when testing A_i against control ($i = 1, 2$), and "CI Width A_i" and "Variance A_i" refers to the width of the confidence interval and variance, respectively, for the treatment effect of arm A_i against control.

Design	r_1	r_2	Allocation	Power *A1*	Power *A2*	CI Width *A1*	CI Width *A2*
1-period	1.000	0.000	one	0.799	0.796	1.105	1.106
	1.000	0.000	opt	0.805	0.805	1.099	1.099
	1.000	0.000	sqrt	0.805	0.804	1.100	1.099
2-period	0.250	0.750	one	0.844	0.666	1.006	1.006
	0.250	0.750	opt	0.772	0.759	0.997	0.997
	0.250	0.750	sqrt	0.852	0.683	0.997	0.997
3-period	0.337	0.326	one	0.717	0.721	1.027	1.151
	0.337	0.326	opt	0.724	0.724	1.085	1.096
	0.337	0.326	sqrt	0.726	0.725	1.025	1.150
3-period	0.337	0.446	one	0.782	0.687	0.947	1.173
	0.337	0.446	opt	0.737	0.727	1.038	1.055
	0.337	0.446	sqrt	0.786	0.688	0.938	1.157

Table 7: Type 1 error (T1E) rates for each design according to the allocation strategy for a trial with stepwise time trends (see (2.h) for the data generation). Here r_1 and r_2 are the proportion of patients allocated to periods 1 and 2, respectively; "one" denotes one-to-one allocation, "opt" denotes optimal allocation and "sqrt" denotes the square root of k allocation; "T1E A_i" is the estimated type 1 error when testing A_i against control ($i = 1, 2$); "CI Width A_i" and "Variance A_i" refers to the width of the confidence interval and variance, respectively, for the treatment effect of arm A_i against control.

Design	r_1	r_2	Allocation	T1E *A1*	T1E *A2*	CI Width *A1*	CI Width *A2*
1-period	1.000	0.000	one	0.025	0.024	1.105	1.105
	1.000	0.000	opt	0.025	0.025	1.100	1.100
	1.000	0.000	sqrt	0.025	0.025	1.099	1.100
2-period	0.250	0.750	one	0.026	0.024	1.006	1.006
	0.250	0.750	opt	0.025	0.025	0.997	0.997
	0.250	0.750	sqrt	0.025	0.026	0.998	0.998
3-period	0.337	0.326	one	0.024	0.024	1.027	1.151
	0.337	0.326	opt	0.025	0.025	1.084	1.095
	0.337	0.326	sqrt	0.025	0.024	1.026	1.150
3-period	0.337	0.446	one	0.025	0.025	0.947	1.173
	0.337	0.446	opt	0.025	0.025	1.038	1.055
	0.337	0.446	sqrt	0.025	0.025	0.939	1.157

C Additional optimisation results

C.1 Optimal solutions under unequal variances across arms

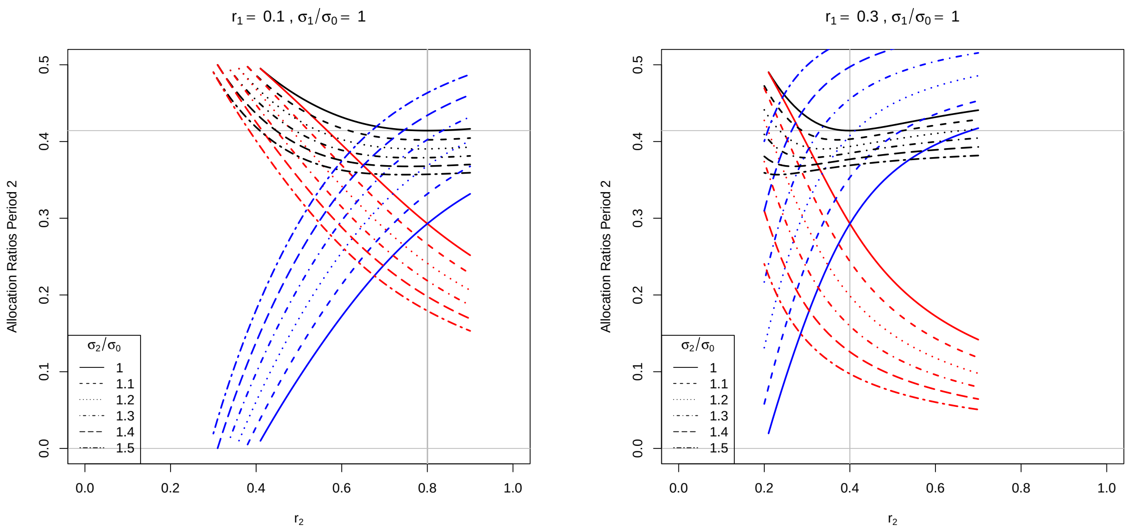

Figure 1: Optimal allocation probabilities $p_{i,2}, i = 0, 1, 2$ in period 2 as function of r_2 and different r_1 for trials with three-periods. Black lines: $p_{0,2}$, red line $p_{1,2}$, blue line $p_{2,2}$. The vertical line at $r_2 = 1 - 2 * r_1$ indicates where equal allocation between treatments 1 and 2 is optimal for the design with concurrent controls only. There the optimal allocation ratio to control is $p_{0,2} = 1/(1 + \sqrt{2})$ (indicated by the horizontal gray line). Solid lines correspond to optimal allocations in trials minising the sum of the variances and dashed lines correspond to optimal allocations in trials minimising the maximum of the variances.

C.2 Optimal solutions when minimising the sum of variances

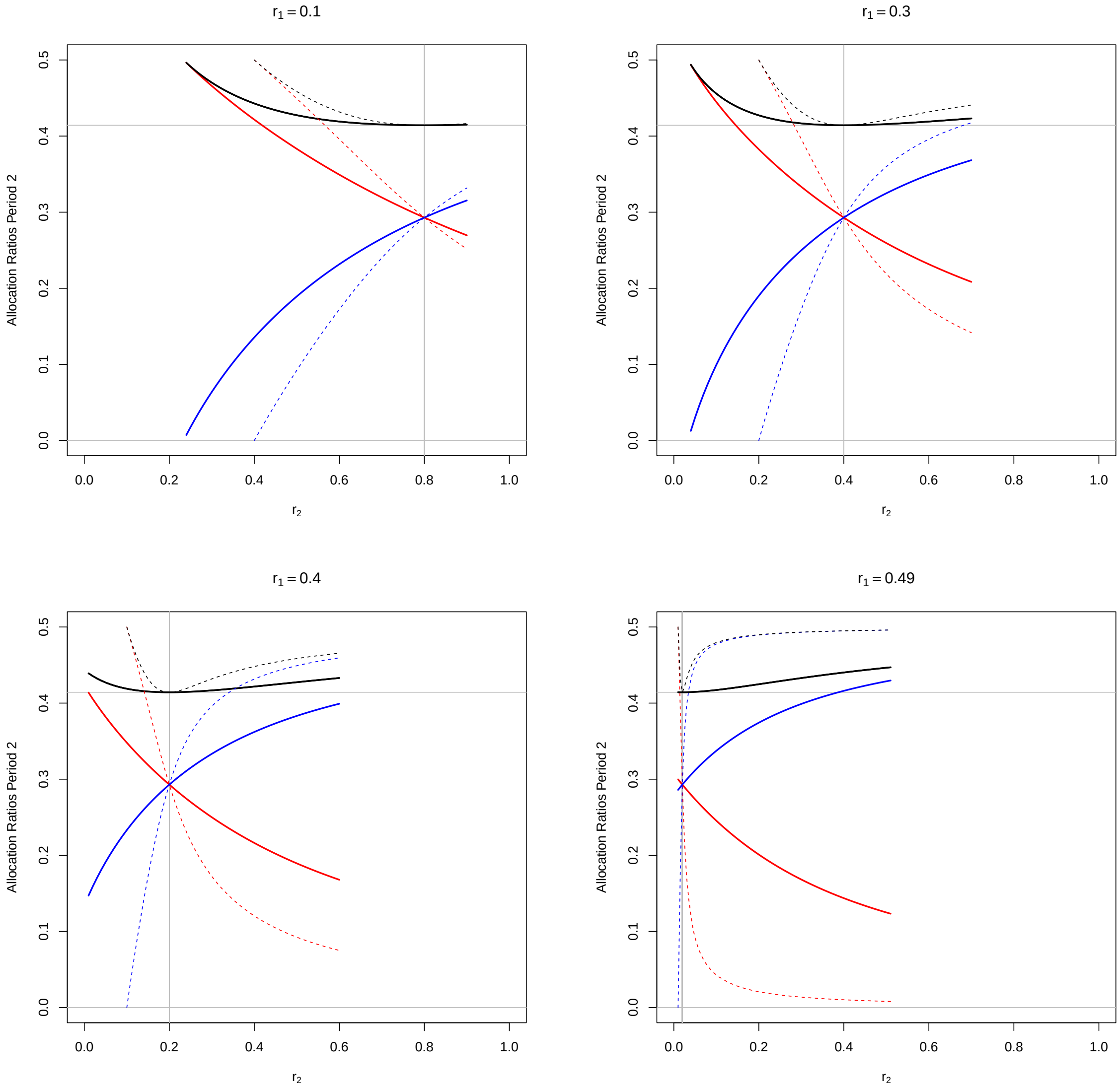

Figure 2: Optimal allocation probabilities $p_{i,2}, i = 0, 1, 2$ in period 2 as function of r_2 and different r_1 for trials with three-periods. Black lines: $p_{0,2}$, red line $p_{1,2}$, blue line $p_{2,2}$. The vertical line at $r_2 = 1 - 2 * r_1$ indicates where equal allocation between treatments 1 and 2 is optimal for the design with concurrent controls only. There the optimal allocation ratio to control is $p_{0,2} = 1/(1 + \sqrt{2})$ (indicated by the horizontal gray line). Solid lines correspond to optimal allocations in trials minising the sum of the variances and dashed lines correspond to optimal allocations in trials minimising the maximum of the variances.

D Mathematica files

The following Mathematica notebook files are now displayed:

- suppmat-CC.nb: Computation on designs with concurrent controls only.
- suppmat-NCC-case2.nb: Computations on case 2 using non-concurrent controls.
- suppmat-NCC-case3.nb: Computations on case 3 using non-concurrent controls.
- suppmat-optimisation-unequalvar.nb: Computations on designs with concurrent controls only. Optimisation under unequal variances.
- suppmat-optimisation-CC-sumvars.nb: Computations on designs with concurrent controls only. Optimisation of the sum of variances.

Mathematica and R code files are also available at `https://github.com/MartaBofillRoig/Allocation`.

Supplementary Material of “Optimal allocation strategies in platform trials”

Design with concurrent controls only

In this file, we provide the derivation of the optimal allocation for trials utilising concurrent controls only. In what follows, we focus on determining the optimal solutions for the Case 3 described in the paper.

Note that Case 2 is a particular case of Case 3 when consider r3=0. By inspection of Figure 3 in the manuscript, one can see the decrease in variance compared to separate trials with respect to r2, and that the maximum variance reduction occurs for r1+r2=1, and thus for a two-period trial.

Case 3 (Section 3.3)

Set and simplify conditions

```
In[1]:= subst = {n11 → r1 * N / 2 , n01 → r1 * N / 2 ,
           n12 → r2 * N - n02 - n22 , n03 → r3 * N / 2, n23 → r3 * N / 2};
        substp = {n12 → r2 * N * p12, n22 → r2 * N * p22, n02 → r2 * N * p02, r3 → 1 - r1 - r2};
```

Define terms to optimise.

Note: *sigma*term1^(-1)/N* is the variance of the estimator of effect 1 (analogously *sigma*term2^(-1)/N* for effect 2). But since sigma and N are fixed, we simply work on term1 and term2 expressions.

```
In[3]:= term1 =
         FullSimplify[((n11 * n01 / (n11 + n01)) + (n12 * n02 / (n12 + n02))) / N /. subst /. substp]
```

Out[3]= $$\frac{r1}{4}+\frac{p02\ (-1+p02+p22)\ r2}{-1+p22}$$

```
In[4]:= term2 =
         FullSimplify[(n22 * n02 / (n22 + n02) / N) + (n23 * n03 / (n23 + n03) / N) /. subst /. substp]
```

Out[4]= $$\frac{1}{4}\ (1-r1-r2)+\frac{p02\ p22\ r2}{p02+p22}$$

```
In[5]:= constr = FullSimplify[term1 - term2]
```

Out[5]= $$\frac{1}{4}\left(-1+2\ r1+r2+\frac{4\ p02\ (-1+p02+p22)\ r2}{-1+p22}-\frac{4\ p02\ p22\ r2}{p02+p22}\right)$$

In[6]:=
```
e1 = Solve[D[term2, p02] == l D[constr, p02], l]
e2 = Solve[D[term2, p22] == l D[constr, p22], l]
```

Out[6]= $$\left\{\left\{l \to \frac{4\left(\frac{p02\,p22\,r2}{(p02+p22)^2} - \frac{p22\,r2}{p02+p22}\right)}{-\frac{4\,p02\,r2}{-1+p22} - \frac{4\,(-1+p02+p22)\,r2}{-1+p22} - \frac{4\,p02\,p22\,r2}{(p02+p22)^2} + \frac{4\,p22\,r2}{p02+p22}}\right\}\right\}$$

Out[7]= $$\left\{\left\{l \to \frac{4\left(\frac{p02\,p22\,r2}{(p02+p22)^2} - \frac{p02\,r2}{p02+p22}\right)}{-\frac{4\,p02\,r2}{-1+p22} + \frac{4\,p02\,(-1+p02+p22)\,r2}{(-1+p22)^2} - \frac{4\,p02\,p22\,r2}{(p02+p22)^2} + \frac{4\,p02\,r2}{p02+p22}}\right\}\right\}$$

In[8]:=
```
e3 = e1[[1]][[1]][[2]] == e2[[1]][[1]][[2]]
```

Out[8]= $$\frac{4\left(\frac{p02\,p22\,r2}{(p02+p22)^2} - \frac{p22\,r2}{p02+p22}\right)}{-\frac{4\,p02\,r2}{-1+p22} - \frac{4\,(-1+p02+p22)\,r2}{-1+p22} - \frac{4\,p02\,p22\,r2}{(p02+p22)^2} + \frac{4\,p22\,r2}{p02+p22}} == \frac{4\left(\frac{p02\,p22\,r2}{(p02+p22)^2} - \frac{p02\,r2}{p02+p22}\right)}{-\frac{4\,p02\,r2}{-1+p22} + \frac{4\,p02\,(-1+p02+p22)\,r2}{(-1+p22)^2} - \frac{4\,p02\,p22\,r2}{(p02+p22)^2} + \frac{4\,p02\,r2}{p02+p22}}$$

In[9]:=
```
sol = Solve[e3, {p02}][[3]]
```

Out[9]= $$\left\{p02 \to \frac{-1 + 2\,p22 - 2\,p22^2}{2\,(-1 + p22)}\right\}$$

The solution corresponds to the optimal allocation for p02 (Section 3.3 in the paper):

In[10]:=
```
FullSimplify[sol]
```

Out[10]= $$\left\{p02 \to \frac{1}{2 - 2\,p22} - p22\right\}$$

Where the optimal solution for p22 can be obtained numerically by solving the following equation:

In[11]:=
```
eq2 = Assuming[{r2 + r3 > 1 / 2, r2 + r3 < 1, p22 > 0, p22 < 1},
  FullSimplify[(term1 - term2) /. sol]]
```

Out[11]= $$\frac{1}{4}\left(-1 + 2\,r1 + \frac{(-2 + p22\,(11 + p22\,(-29 + p22\,(49 - 4\,p22\,(13 + 2\,(-4 + p22)\,p22)))))\,r2}{(-1 + p22)^3}\right)$$

In[12]:=
```
soleq = Assuming[{p22 > 0, p22 < 1}, FullSimplify[Solve[(eq2 == 0), r2]]]
```

Out[12]= $$\left\{\left\{r2 \to \frac{(-1 + p22)^3\,(-1 + 2\,r1)}{(-1 + 2\,p22)\,(-2 + p22\,(7 + p22\,(-15 + p22\,(19 + 2\,p22\,(-7 + 2\,p22)))))}\right\}\right\}$$

Supplementary Material of “Optimal allocation strategies in platform trials”

Design with concurrent and non-concurrent controls - Case 2

In this file, we provide the derivation of the optimal allocation for trials utilising concurrent and non-concurrent controls. Next, we focus on determining the optimal solutions for the Case 2 described in the paper.

For simplicity of calculation, in this file we start optimising using a different parametrization. We consider: r_{i,s} = n_{i,s} /N_s, where as before n_{i,s} is the sample size for arm i in the period s, and N_s is the total sample size in period s. At the end of the document we express the solutions in terms of p_{i,s} as in the paper.

Set conditions

In[1]:=
```
subst = {r11 → r1 / 2 , r01 → r1 / 2 , r02 → (1 - r1) - r12 - r22 };
```

In[2]:=
```
substp = {r12 → r2 p12, r22 → r2 p22, r02 → r2 p02, r3 → 1 - r1 - r2};
```

Define terms to optimise.

In[3]:=
```
term1 = FullSimplify[(r11 * r01 / (r11 + r01)) + (r12 * r02 / (r12 + r02)) /. subst]
```

Out[3]=
$$\frac{r1}{4} + r12 + \frac{r12^2}{-1 + r1 + r22}$$

In[4]:=
```
term2 = FullSimplify[
  (1 / r22 + 1 / r02 - ((1 / r02) ^2 / (1 / r01 + 1 / r02 + 1 / r11 + 1 / r12))) ^ (-1) /. subst]
```

Out[4]=
$$\frac{r22\left(r1^2 + 4\,r12\,(-1 + r12 + r22) + r1\,(-1 + 4\,r12 + r22)\right)}{r1^2 + 4\,(-1 + r12)\,r12 + r1\,(-1 + 4\,r12)}$$

In[5]:=
```
sol = Solve[term1 == term2, r12] [[3]]
```

Out[5]=
$$\left\{r12 \to \frac{1}{2}\left(1 - r1 - \sqrt{1 - r1 - 4\,r22 + 4\,r1\,r22 + 4\,r22^2}\right)\right\}$$

In[6]:=
```
term3 = Simplify[term1 /. sol]
```

Out[6]=
$$\frac{r22\left(-2 + 3\,r1 + 4\,r22 - 2\sqrt{(1 - 2\,r22)^2 + r1\,(-1 + 4\,r22)}\right)}{4\,(-1 + r1 + r22)}$$

In[7]:=
```
derivative = FullSimplify[D[term3, r22]]
```

Out[7]=

$$\frac{1}{4\,(-1+r1+r22)^{2}}\left(r22\,(-1+r1+r22)\left(4-\frac{4\,(-1+r1+2\,r22)}{\sqrt{(1-2\,r22)^{2}+r1\,(-1+4\,r22)}}\right)-r22\left(-2+3\,r1+4\,r22-2\sqrt{(1-2\,r22)^{2}+r1\,(-1+4\,r22)}\right)+(-1+r1+r22)\left(-2+3\,r1+4\,r22-2\sqrt{(1-2\,r22)^{2}+r1\,(-1+4\,r22)}\right)\right)$$

In[8]:=
```
sol2 = Solve[derivative == 0, r22];
```

Solve: There may be values of the parameters for which some or all solutions are not valid.

In[9]:=
```
sol22 = Assuming[{r1 > 0, r1 < 1 / 2}, Simplify[sol2[[3]]]]
```

Out[9]=

$$\left\{r22\to 1-r1+\frac{1}{4\sqrt{3}}\left(\sqrt{\left(\left((-1+r1)\left(9\,r1^{2}+6\,r1\left(-4+\left(8+36\,r1-108\,r1^{2}+27\,r1^{3}+6\sqrt{3}\sqrt{r1\,(16-72\,r1+108\,r1^{2}-27\,r1^{3})}\right)^{1/3}\right)+\left(-2+\left(8+36\,r1-108\,r1^{2}+27\,r1^{3}+6\sqrt{3}\sqrt{r1\,(16-72\,r1+108\,r1^{2}-27\,r1^{3})}\right)^{1/3}\right)^{2}\right)\right)\Big/\left(8+36\,r1-108\,r1^{2}+27\,r1^{3}+6\sqrt{3}\sqrt{r1\,(16-72\,r1+108\,r1^{2}-27\,r1^{3})}\right)^{1/3}\right)}-\frac{1}{2}\sqrt{\left(-\frac{22}{3}+8\,(-1+r1)^{2}+\frac{43\,r1}{3}-7\,r1^{2}-\frac{(-1+r1)\,(4-24\,r1+9\,r1^{2})}{12\left(8+36\,r1-108\,r1^{2}+27\,r1^{3}+6\sqrt{3}\sqrt{r1\,(16-72\,r1+108\,r1^{2}-27\,r1^{3})}\right)^{1/3}}-\frac{1}{12}(-1+r1)\left(8+36\,r1-108\,r1^{2}+27\,r1^{3}+6\sqrt{3}\sqrt{r1\,(16-72\,r1+108\,r1^{2}-27\,r1^{3})}\right)^{1/3}+\left(\sqrt{3}\,(-1+r1)^{2}\,r1\left(8+36\,r1-108\,r1^{2}+27\,r1^{3}+6\sqrt{3}\sqrt{r1\,(16-72\,r1+108\,r1^{2}-27\,r1^{3})}\right)^{1/6}\right)\Big/\left(\sqrt{\left((-1+r1)\left(9\,r1^{2}+6\,r1\left(-4+\left(8+36\,r1-108\,r1^{2}+27\,r1^{3}+6\sqrt{3}\sqrt{r1\,(16-72\,r1+108\,r1^{2}-27\,r1^{3})}\right)^{1/3}\right)+\left(-2+\left(8+36\,r1-108\,r1^{2}+27\,r1^{3}+6\sqrt{3}\sqrt{r1\,(16-72\,r1+108\,r1^{2}-27\,r1^{3})}\right)^{1/3}\right)^{2}\right)\right)}\right)\right)}\right)\right\}$$

In[10]:=
```
CForm[sol22[[1]][[2]]]
```

Out[10]//CForm=
```
1 - r1 + Sqrt(((-1 + r1)*(9*Power(r1,2) + 6*r1*(-4 + Power(8 + 36*r1 - 108*Power(r1,2)
            0.3333333333333333)) + Power(-2 + Power(8 + 36*r1 - 108*Power(r1,2) + 27*
           0.3333333333333333),2)))/Power(8 + 36*r1 - 108*Power(r1,2) + 27*Power(r1,3
    (4.*Sqrt(3)) - Sqrt(-7.333333333333333 + 8*Power(-1 + r1,2) + (43*r1)/3. - 7*Power
      ((-1 + r1)*(4 - 24*r1 + 9*Power(r1,2)))/
       (12.*Power(8 + 36*r1 - 108*Power(r1,2) + 27*Power(r1,3) + 6*Sqrt(3)*Sqrt(r1*(16
      ((-1 + r1)*Power(8 + 36*r1 - 108*Power(r1,2) + 27*Power(r1,3) + 6*Sqrt(3)*Sqrt(r
      (Sqrt(3)*Power(-1 + r1,2)*r1*Power(8 + 36*r1 - 108*Power(r1,2) + 27*Power(r1,3)
       Sqrt((-1 + r1)*(9*Power(r1,2) + 6*r1*(-4 + Power(8 + 36*r1 - 108*Power(r1,2) +
             0.3333333333333333)) + Power(-2 + Power(8 + 36*r1 - 108*Power(r1,2) + 27
            0.3333333333333333),2))))/2.
```

In[11]:=
```
sol22 /. {r1 → 0.3, r2 → 0.7}
```

Out[11]= {r22 → 0.302281}

Next, we simplify the solution by defining terms a and b.

In[12]:=
```
sol22a = FullSimplify[sol22 /. {FullSimplify[
    (8 + 36 r1 - 108 r1^2 + 27 r1^3 + 6 Sqrt[3] Sqrt[r1 (16 - 72 r1 + 108 r1^2 - 27 r1^3)])^(1/3)] → a }]
```

Out[12]=

$$\Bigg\{r22 \to 1 - r1 + \frac{1}{4\sqrt{3}}\left(\sqrt{\left(\left((-1+r1)\left(9\,r1^2 + 6\,r1\left(-4+\left(8+9\,r1\,(4+3\,(-4+r1)\,r1)+6\sqrt{3}\sqrt{r1\,(16-9\,r1\,(8+3\,(-4+r1)\,r1))}\right)^{1/3}\right)+\left(-2+\left(8+9\,r1\,(4+3\,(-4+r1)\,r1)+6\sqrt{3}\sqrt{r1\,(16-9\,r1\,(8+3\,(-4+r1)\,r1))}\right)^{1/3}\right)^2\right)\right)\Big/\left(8+9\,r1\,(4+3\,(-4+r1)\,r1)+6\sqrt{3}\sqrt{r1\,(16-9\,r1\,(8+3\,(-4+r1)\,r1))}\right)^{1/3}\right)}\right) - \frac{1}{2}\sqrt{\left(-\frac{22}{3}+8\,(-1+r1)^2+\frac{43\,r1}{3}-7\,r1^2-\frac{(-1+r1)\,(4+3\,r1\,(-8+3\,r1))}{12\left(8+9\,r1\,(4+3\,(-4+r1)\,r1)+6\sqrt{3}\sqrt{r1\,(16-9\,r1\,(8+3\,(-4+r1)\,r1))}\right)^{1/3}}-\frac{1}{12}(-1+r1)\left(8+9\,r1\,(4+3\,(-4+r1)\,r1)+6\sqrt{3}\sqrt{r1\,(16-9\,r1\,(8+3\,(-4+r1)\,r1))}\right)^{1/3}+\left(\sqrt{3}\,(-1+r1)^2\,r1\left(8+9\,r1\,(4+3\,(-4+r1)\,r1)+6\sqrt{3}\sqrt{r1\,(16-9\,r1\,(8+3\,(-4+r1)\,r1))}\right)^{1/6}\right)\Big/\left(\sqrt{\left((-1+r1)\left(9\,r1^2+6\,r1\left(-4+\left(8+9\,r1\,(4+3\,(-4+r1)\,r1)+6\sqrt{3}\sqrt{r1\,(16-9\,r1\,(8+3\,(-4+r1)\,r1))}\right)^{1/3}\right)+\left(-2+\left(8+9\,r1\,(4+3\,(-4+r1)\,r1)+6\sqrt{3}\sqrt{r1\,(16-9\,r1\,(8+3\,(-4+r1)\,r1))}\right)^{1/3}\right)^2\right)\right)}\right)\right)}\Bigg\}$$

In[13]:=
```
FullSimplify[(8 + 9 r1 (4 + 3 (-4 + r1) r1) + 6 Sqrt[3] Sqrt[r1 (16 - 9 r1 (8 + 3 (-4 + r1) r1))])^(1/3) /
  (8 + 36 r1 - 108 r1^2 + 27 r1^3 + 6 Sqrt[3] Sqrt[r1 (16 - 72 r1 + 108 r1^2 - 27 r1^3)])^(1/3)]
```

Out[13]= 1

In[14]:=
```
sol22a /. {(8 + 9 r1 (4 + 3 (-4 + r1) r1) + 6 √3 √(r1 (16 - 9 r1 (8 + 3 (-4 + r1) r1))))^(1/3) → a}
```

Out[14]=

$$\left\{r22 \to 1 - r1 + \frac{\sqrt{\frac{(-1+r1)\left((-2+a)^2+6(-4+a)\,r1+9\,r1^2\right)}{\left(8+9\,r1\,(4+3(-4+r1)\,r1)+6\sqrt{3}\sqrt{r1\,(16-9\,r1\,(8+3(-4+r1)\,r1))}\right)^{1/3}}}}{4\sqrt{3}} - \frac{1}{2}\sqrt{\left(-\frac{22}{3} - \frac{1}{12}a(-1+r1) + 8(-1+r1)^2 + \frac{43\,r1}{3} - 7\,r1^2 - \frac{(-1+r1)(4+3\,r1(-8+3\,r1))}{12\left(8+9\,r1\,(4+3(-4+r1)\,r1)+6\sqrt{3}\sqrt{r1\,(16-9\,r1\,(8+3(-4+r1)\,r1))}\right)^{1/3}} + \left(\sqrt{3}\,(-1+r1)^2\,r1\left(8+9\,r1\,(4+3(-4+r1)\,r1)+6\sqrt{3}\sqrt{r1\,(16-9\,r1\,(8+3(-4+r1)\,r1))}\right)^{1/6}\right) \Big/ \left(\sqrt{(-1+r1)\left((-2+a)^2+6(-4+a)\,r1+9\,r1^2\right)}\right)\right)}\right\}$$

In[15]:=
```
r22a = 1 - r1 + (√(((-1 + r1) ((-2 + a)^2 + 6 (-4 + a) r1 + 9 r1^2)) / a)) / (4 × √3) -
  (1 / 2) √(- (22 / 3) - (1 / 12) a (-1 + r1) + 8 (-1 + r1)^2 +
     (43 r1) / 3 - 7 r1^2 - ((-1 + r1) (4 + 3 r1 (-8 + 3 r1))) / (12 a) +
     (√3 (-1 + r1)^2 r1 (a^3)^(1/6)) / (√((-1 + r1) ((-2 + a)^2 + 6 (-4 + a) r1 + 9 r1^2))))
```

Out[15]=

$$1 - r1 + \frac{\sqrt{\frac{(-1+r1)\left((-2+a)^2+6(-4+a)\,r1+9\,r1^2\right)}{a}}}{4\sqrt{3}} - \frac{1}{2}\sqrt{\left(-\frac{22}{3} - \frac{1}{12}a(-1+r1) + 8(-1+r1)^2 + \frac{43\,r1}{3} - 7\,r1^2 + \frac{\sqrt{3}\,(a^3)^{1/6}(-1+r1)^2\,r1}{\sqrt{(-1+r1)\left((-2+a)^2+6(-4+a)\,r1+9\,r1^2\right)}} - \frac{(-1+r1)(4+3\,r1(-8+3\,r1))}{12\,a}\right)}$$

In[16]:=
```
CForm[r22a]
```

Out[16]//CForm=
```
1 - r1 + Sqrt(((-1 + r1)*(Power(-2 + a,2) + 6*(-4 + a)*r1 + 9*Power(r1,2)))/a)/(4.*Sq
   Sqrt(-7.333333333333333 - (a*(-1 + r1))/12. + 8*Power(-1 + r1,2) + (43*r1)/3. - 7*P
      (Sqrt(3)*Power(Power(a,3),0.16666666666666666)*Power(-1 + r1,2)*r1)/Sqrt((-1 + r
   2.
```

In[17]:= **FullSimplify[r22a]**

Out[17]=
$$1-r1+\frac{\sqrt{\frac{(-1+r1)\left((-2+a)^2+6(-4+a)\,r1+9\,r1^2\right)}{a}}}{4\sqrt{3}}-\frac{1}{2}\sqrt{\left(-\frac{22}{3}-\frac{1}{12}a\,(-1+r1)+8\,(-1+r1)^2+\frac{43\,r1}{3}-7\,r1^2+\frac{\sqrt{3}\,\left(a^3\right)^{1/6}(-1+r1)^2\,r1}{\sqrt{(-1+r1)\left((-2+a)^2+6\,(-4+a)\,r1+9\,r1^2\right)}}-\frac{(-1+r1)\,(4+3\,r1\,(-8+3\,r1))}{12\,a}\right)}$$

In[18]:= **sol22ab = FullSimplify[r22a /. {(-2 + a)^2 + 6 (-4 + a) r1 + 9 r1^2 → b}]**

Out[18]=
$$\frac{1}{12}\left(12+\sqrt{3}\sqrt{\frac{b\,(-1+r1)}{a}}-12\,r1-\sqrt{3}\sqrt{-\frac{(-1+r1)\left(-12\sqrt{3}\,a\left(a^3\right)^{1/6}\sqrt{b\,(-1+r1)}\,r1+b\left(4+8\,a+a^2-12\,(2+a)\,r1+9\,r1^2\right)\right)}{a\,b}}\right)$$

In[19]:= **sol22abs = FullSimplify[PowerExpand[r22a /. {(-2 + a)^2 + 6 (-4 + a) r1 + 9 r1^2 → b}]]**

Out[19]=
$$\frac{1}{12}\left(12+\frac{\sqrt{3}\sqrt{b}\sqrt{-1+r1}}{\sqrt{a}}-12\,r1-\sqrt{3}\sqrt{-\frac{(-1+r1)\left(-12\sqrt{3}\,a^{3/2}\sqrt{-1+r1}\,r1+\sqrt{b}\left(4+8\,a+a^2-12\,(2+a)\,r1+9\,r1^2\right)\right)}{a\sqrt{b}}}\right)$$

In[20]:= **CForm[sol22ab]**

Out[20]//CForm=
```
(12 + Sqrt(3)*Sqrt((b*(-1 + r1))/a) - 12*r1 - Sqrt(3)*Sqrt(-(((-1 + r1)*(-12*Sqrt(3)*
         b*(4 + 8*a + Power(a,2) - 12*(2 + a)*r1 + 9*Power(r1,2))))/(a*b))))/12.
```

We compute the solution in terms of p22 by taking into account that p22=r22/(1-r1), as 1-r1=r2 in this case.

In[21]:= **solp22 = FullSimplify[sol22ab / (1 - r1)]**

Out[21]=
$$\frac{-12-\sqrt{3}\sqrt{\frac{b\,(-1+r1)}{a}}+12\,r1+\sqrt{3}\sqrt{-\frac{(-1+r1)\left(-12\sqrt{3}\,a\left(a^3\right)^{1/6}\sqrt{b\,(-1+r1)}\,r1+b\left(4+8\,a+a^2-12\,(2+a)\,r1+9\,r1^2\right)\right)}{a\,b}}}{12\,(-1+r1)}$$

In[22]:= **CForm[solp22]**

Out[22]//CForm=
```
(-12 - Sqrt(3)*Sqrt((b*(-1 + r1))/a) + 12*r1 + Sqrt(3)*Sqrt(-(((-1 + r1)*(-12*Sqrt(3)
         b*(4 + 8*a + Power(a,2) - 12*(2 + a)*r1 + 9*Power(r1,2))))/(a*b))))/(12.
```

Simplifying solution p22

First note that a>0 and b<0

In[23]:= $\left(8+9\,r1\,(4+3\,(-4+r1)\,r1)+6\sqrt{3}\,\sqrt{r1\,(16-9\,r1\,(8+3\,(-4+r1)\,r1))}\right)^{1/3}$ `/. {r1 → 0.3}`

Out[23]= 2.72751

In[24]:= $(-2+a)^2+6\,(-4+a)\,r1+9\,r1^2$ `/. {r1 → 0.3, a → 2.727510816380422}`

Out[24]= -0.951209

In[25]:= $$\text{FullSimplify}\left[\frac{-12+12\,r1}{12\,(-1+r1)}\right]+\text{FullSimplify}\left[\frac{-\sqrt{3}\,\sqrt{b\,(-1+r1)/a}+\sqrt{3}\,\sqrt{-\frac{(-1+r1)\left(-12\sqrt{3}\,a\,(a^3)^{1/6}\sqrt{b\,(-1+r1)}\,r1+b\left(4+8\,a+a^2-12\,(2+a)\,r1+9\,r1^2\right)\right)}{a\,b}}}{12\,(-1+r1)}\right]$$

Out[25]= $$1+\frac{-\sqrt{\frac{b\,(-1+r1)}{a}}+\sqrt{-\frac{(-1+r1)\left(-12\sqrt{3}\,a\,(a^3)^{1/6}\sqrt{b\,(-1+r1)}\,r1+b\left(4+8\,a+a^2-12\,(2+a)\,r1+9\,r1^2\right)\right)}{a\,b}}}{4\sqrt{3}\,(-1+r1)}$$

In[26]:= solp22s = Assuming[{a > 0, b < 0, r1 < 1, r1 > 0},

$$\text{FullSimplify}\left[1+\frac{-\sqrt{\frac{b\,(-1+r1)}{a}}+\sqrt{-\frac{(-1+r1)\left(-12\sqrt{3}\,a\,(a^3)^{1/6}\sqrt{b\,(-1+r1)}\,r1+b\left(4+8\,a+a^2-12\,(2+a)\,r1+9\,r1^2\right)\right)}{a\,b}}}{4\sqrt{3}\,(-1+r1)}\right]\Bigg]$$

Out[26]= $$1+\frac{-\sqrt{\frac{b\,(-1+r1)}{a}}+\sqrt{-\frac{(-1+r1)\left(-12\sqrt{3}\,\sqrt{a^3\,b\,(-1+r1)}\,r1+b\left(4+8\,a+a^2-12\,(2+a)\,r1+9\,r1^2\right)\right)}{a\,b}}}{4\sqrt{3}\,(-1+r1)}$$

In[27]:= $$\text{solp22s2} = 1+\frac{\sqrt{-b}-\sqrt{12\sqrt{3}\,\sqrt{a^3\,b\hat{}(-1)\,(-1+r1)}\,r1+\left(4+8\,a+a^2-12\,(2+a)\,r1+9\,r1^2\right)}}{4\sqrt{3\,a\,(1-r1)}}$$

Out[27]= $$1+\frac{\sqrt{-b}-\sqrt{4+8\,a+a^2-12\,(2+a)\,r1+12\sqrt{3}\,\sqrt{\frac{a^3\,(-1+r1)}{b}}\,r1+9\,r1^2}}{4\sqrt{3}\,\sqrt{a\,(1-r1)}}$$

In[28]:= CForm[solp22s2]

Out[28]//CForm=

```
1 + (Sqrt(-b) - Sqrt(4 + 8*a + Power(a,2) - 12*(2 + a)*r1 + 12*Sqrt(3)*Sqrt((Power(a,3
```

In[29]:= solp22s /. {r1 → 0.3, a → 2.727510816380422`, b → -0.9512085425647322`}

Out[29]= 0.43183

In[30]:= solp22s2 /. {r1 → 0.3, a → 2.727510816380422`, b → -0.9512085425647322`}

Out[30]= 0.43183

In[31]:= Assuming[{a > 0, b < 0, r1 < 1, r1 > 0}, FullSimplify[solp22s / solp22]]

Out[31]= 1

Finally, to get the solution r12 (and hence p12), one needs to use the solution obtained for r22 and substitute it in the expression for r12 (***sol***).

In[32]:= **sol**

Out[32]= $\left\{r12 \to \frac{1}{2}\left(1 - r1 - \sqrt{1 - r1 - 4\,r22 + 4\,r1\,r22 + 4\,r22^2}\right)\right\}$

In[33]:= **solp22 = FullSimplify[sol⟦1⟧⟦2⟧ / (1 - r1) /. {r22 → p22 * (1 - r1)}]**

Out[33]= $\frac{-1 + \sqrt{(-1 + 4\,(-1 + p22)\,p22\,(-1 + r1))\,(-1 + r1)} + r1}{2\,(-1 + r1)}$

In[34]:= **solp22s = FullSimplify[solp22 /.**

$$\left\{p22 \to 1 + \frac{\sqrt{-b\,(1 - r1)} - \sqrt{\frac{(1-r1)\,\left(-12\,\sqrt{3}\,a\,\left(a^3\right)^{1/6}\,\sqrt{-b\,(1-r1)}\,r1 + b\,\left(4 + 8\,a + a^2 - 12\,(2+a)\,r1 + 9\,r1^2\right)\right)}{b}}}{4\,\sqrt{3\,a}\,(1 - r1)}\right\}\Big]$$

Out[34]= $$\frac{1}{2} + \frac{1}{2\,(-1 + r1)}\left(\sqrt{\left((-1 + r1)\left(-1 + \frac{1}{12\,\sqrt{3}\,a\,(-1 + r1)}\left(\sqrt{b\,(-1 + r1)} - \sqrt{\left(-\frac{1}{b}(-1 + r1)\left(-12\,\sqrt{3}\,a\,\left(a^3\right)^{1/6}\,\sqrt{b\,(-1 + r1)}\,r1 + b\,\left(4 + 8\,a + a^2 - 12\,(2 + a)\,r1 + 9\,r1^2\right)\right)\right)}\right)\right)\left(-12\,\sqrt{a}\,(-1 + r1) + \sqrt{3}\left(\sqrt{b\,(-1 + r1)} - \sqrt{\left(-\frac{1}{b}(-1 + r1)\left(-12\,\sqrt{3}\,a\,\left(a^3\right)^{1/6}\,\sqrt{b\,(-1 + r1)}\,r1 + b\,\left(4 + 8\,a + a^2 - 12\,(2 + a)\,r1 + 9\,r1^2\right)\right)\right)}\right)\right)\right)}\right)$$

Supplementary Material of “Optimal allocation strategies in platform trials”

Design with concurrent and non-concurrent controls - Case 3

We first define the treatment effect for arm 2 following the expressions presented in the supplementary material (see Section A.2). To do so, we define the matrices A, B, and C, and use equation (1.b) to to obtain point estimates

In[ ]:=
```
nd1 = n01 + n11
nd2 = n02 + n12 + n22
nd3 = n03 + n23
A = {{nd1, 0, 0}, {0, nd2, 0}, {0, 0, nd3}}
B = {{n11, 0}, {n12, n22}, {0, n23}}
Cm = {{n11 + n12, 0}, {0, n22 + n23}}
```

Out[ ]=
```
n01 + n11
```

Out[ ]=
```
n02 + n12 + n22
```

Out[ ]=
```
n03 + n23
```

Out[ ]=
```
{{n01 + n11, 0, 0}, {0, n02 + n12 + n22, 0}, {0, 0, n03 + n23}}
```

Out[ ]=
```
{{n11, 0}, {n12, n22}, {0, n23}}
```

Out[ ]=
```
{{n11 + n12, 0}, {0, n22 + n23}}
```

```
In[]:= M = FullSimplify[Inverse[Cm - Transpose[B].Inverse[A].B]]
Nm = {{n11 * theta11 + n12 * theta12}, {n22 * theta22 + n23 * theta23}}
Collect[FullSimplify[M.Nm][[2]], {theta11, theta12, theta22, theta23}]
w11 = (n11 (n01 + n11) n12 n22 (n03 + n23) ) /
   (n11 n12 (n02 n03 n22 + n03 n22 n23 + n02 (n03 + n22) n23) +
     n01 (n03 n11 n12 n22 + (n03 n11 n12 + n11 n12 n22 + n03 (n11 + n12) n22) n23 +
        n02 (n11 + n12) (n22 n23 + n03 (n22 + n23))))
w12 = ((n01 + n11) n12^2 n22 (n03 + n23) ) /
   (n11 n12 (n02 n03 n22 + n03 n22 n23 + n02 (n03 + n22) n23) +
     n01 (n03 n11 n12 n22 + (n03 n11 n12 + n11 n12 n22 + n03 (n11 + n12) n22) n23 +
        n02 (n11 + n12) (n22 n23 + n03 (n22 + n23))))
w22 =
  (n22 (n11 n12 (n02 + n22) + n01 (n11 n12 + n02 (n11 + n12) + (n11 + n12) n22)) (n03 + n23) ) /
   (n11 n12 (n02 n03 n22 + n03 n22 n23 + n02 (n03 + n22) n23) +
     n01 (n03 n11 n12 n22 + (n03 n11 n12 + n11 n12 n22 + n03 (n11 + n12) n22) n23 +
        n02 (n11 + n12) (n22 n23 + n03 (n22 + n23))))
w23 =
  ((n11 n12 (n02 + n22) + n01 (n11 n12 + n02 (n11 + n12) + (n11 + n12) n22)) n23 (n03 + n23) ) /
   (n11 n12 (n02 n03 n22 + n03 n22 n23 + n02 (n03 + n22) n23) +
     n01 (n03 n11 n12 n22 + (n03 n11 n12 + n11 n12 n22 + n03 (n11 + n12) n22) n23 +
        n02 (n11 + n12) (n22 n23 + n03 (n22 + n23))))
sol = M.Nm
True ===
 FullSimplify[sol[[2]][[1]] == w11 * theta11 + w12 * theta12 + w22 * theta22 + w23 * theta23]

Out[]= {{((n01 + n11) (n03 (n02 + n12) n22 + n03 (n02 + n12) n23 + (n02 + n03 + n12) n22 n23)) /
    (n01 n03 (n11 n12 + n02 (n11 + n12)) n22 +
      n01 (n03 n11 n12 + n11 n12 n22 + n03 (n11 + n12) n22 + n02 (n11 + n12) (n03 + n22)) n23 +
      n11 n12 (n02 n03 n22 + n03 n22 n23 + n02 (n03 + n22) n23)),
   ((n01 + n11) n12 n22 (n03 + n23)) / (n11 n12 (n02 n03 n22 + n03 n22 n23 + n02 (n03 + n22) n23) +
      n01 (n03 n11 n12 n22 + (n03 n11 n12 + n11 n12 n22 + n03 (n11 + n12) n22) n23 +
         n02 (n11 + n12) (n22 n23 + n03 (n22 + n23))))},
  {((n01 + n11) n12 n22 (n03 + n23)) / (n11 n12 (n02 n03 n22 + n03 n22 n23 + n02 (n03 + n22) n23) +
      n01 (n03 n11 n12 n22 + (n03 n11 n12 + n11 n12 n22 + n03 (n11 + n12) n22) n23 +
         n02 (n11 + n12) (n22 n23 + n03 (n22 + n23)))),
   ((n11 n12 (n02 + n22) + n01 (n11 n12 + n02 (n11 + n12) + (n11 + n12) n22)) (n03 + n23)) /
    (n11 n12 (n02 n03 n22 + n03 n22 n23 + n02 (n03 + n22) n23) +
      n01 (n03 n11 n12 n22 + (n03 n11 n12 + n11 n12 n22 + n03 (n11 + n12) n22) n23 +
         n02 (n11 + n12) (n22 n23 + n03 (n22 + n23))))}}

Out[]= {{n11 theta11 + n12 theta12}, {n22 theta22 + n23 theta23}}
```

Out[•]=
```
{(n11 (n01 + n11) n12 n22 (n03 + n23) theta11) /
   (n11 n12 (n02 n03 n22 + n03 n22 n23 + n02 (n03 + n22) n23) +
     n01 (n03 n11 n12 n22 + (n03 n11 n12 + n11 n12 n22 + n03 (n11 + n12) n22) n23 +
         n02 (n11 + n12) (n22 n23 + n03 (n22 + n23)))) +
  ((n01 + n11) n12^2 n22 (n03 + n23) theta12) /
   (n11 n12 (n02 n03 n22 + n03 n22 n23 + n02 (n03 + n22) n23) +
     n01 (n03 n11 n12 n22 + (n03 n11 n12 + n11 n12 n22 + n03 (n11 + n12) n22) n23 +
         n02 (n11 + n12) (n22 n23 + n03 (n22 + n23)))) +
  (n22 (n11 n12 (n02 + n22) + n01 (n11 n12 + n02 (n11 + n12) + (n11 + n12) n22))
      (n03 + n23) theta22) / (n11 n12 (n02 n03 n22 + n03 n22 n23 + n02 (n03 + n22) n23) +
     n01 (n03 n11 n12 n22 + (n03 n11 n12 + n11 n12 n22 + n03 (n11 + n12) n22) n23 +
         n02 (n11 + n12) (n22 n23 + n03 (n22 + n23)))) +
  ((n11 n12 (n02 + n22) + n01 (n11 n12 + n02 (n11 + n12) + (n11 + n12) n22))
     n23 (n03 + n23) theta23) /
   (n11 n12 (n02 n03 n22 + n03 n22 n23 + n02 (n03 + n22) n23) +
     n01 (n03 n11 n12 n22 + (n03 n11 n12 + n11 n12 n22 + n03 (n11 + n12) n22) n23 +
         n02 (n11 + n12) (n22 n23 + n03 (n22 + n23))))}
```

Out[•]=
```
(n11 (n01 + n11) n12 n22 (n03 + n23)) /
 (n11 n12 (n02 n03 n22 + n03 n22 n23 + n02 (n03 + n22) n23) +
   n01 (n03 n11 n12 n22 + (n03 n11 n12 + n11 n12 n22 + n03 (n11 + n12) n22) n23 +
       n02 (n11 + n12) (n22 n23 + n03 (n22 + n23))))
```

Out[•]=
```
((n01 + n11) n12^2 n22 (n03 + n23)) / (n11 n12 (n02 n03 n22 + n03 n22 n23 + n02 (n03 + n22) n23) +
   n01 (n03 n11 n12 n22 + (n03 n11 n12 + n11 n12 n22 + n03 (n11 + n12) n22) n23 +
       n02 (n11 + n12) (n22 n23 + n03 (n22 + n23))))
```

Out[•]=
```
(n22 (n11 n12 (n02 + n22) + n01 (n11 n12 + n02 (n11 + n12) + (n11 + n12) n22)) (n03 + n23)) /
 (n11 n12 (n02 n03 n22 + n03 n22 n23 + n02 (n03 + n22) n23) +
   n01 (n03 n11 n12 n22 + (n03 n11 n12 + n11 n12 n22 + n03 (n11 + n12) n22) n23 +
       n02 (n11 + n12) (n22 n23 + n03 (n22 + n23))))
```

Out[•]=
```
((n11 n12 (n02 + n22) + n01 (n11 n12 + n02 (n11 + n12) + (n11 + n12) n22)) n23 (n03 + n23)) /
 (n11 n12 (n02 n03 n22 + n03 n22 n23 + n02 (n03 + n22) n23) +
   n01 (n03 n11 n12 n22 + (n03 n11 n12 + n11 n12 n22 + n03 (n11 + n12) n22) n23 +
       n02 (n11 + n12) (n22 n23 + n03 (n22 + n23))))
```

```
Out[•]= {{((n01 + n11) (n03 (n02 + n12) n22 + n03 (n02 + n12) n23 + (n02 + n03 + n12) n22 n23)
           (n11 theta11 + n12 theta12)) / (n01 n03 (n11 n12 + n02 (n11 + n12)) n22 +
          n01 (n03 n11 n12 + n11 n12 n22 + n03 (n11 + n12) n22 + n02 (n11 + n12) (n03 + n22)) n23 +
          n11 n12 (n02 n03 n22 + n03 n22 n23 + n02 (n03 + n22) n23)) +
       ((n01 + n11) n12 n22 (n03 + n23) (n22 theta22 + n23 theta23)) /
        (n11 n12 (n02 n03 n22 + n03 n22 n23 + n02 (n03 + n22) n23) +
          n01 (n03 n11 n12 n22 + (n03 n11 n12 + n11 n12 n22 + n03 (n11 + n12) n22) n23 +
              n02 (n11 + n12) (n22 n23 + n03 (n22 + n23))))},
     {((n01 + n11) n12 n22 (n03 + n23) (n11 theta11 + n12 theta12)) /
        (n11 n12 (n02 n03 n22 + n03 n22 n23 + n02 (n03 + n22) n23) +
          n01 (n03 n11 n12 n22 + (n03 n11 n12 + n11 n12 n22 + n03 (n11 + n12) n22) n23 +
              n02 (n11 + n12) (n22 n23 + n03 (n22 + n23)))) +
       ((n11 n12 (n02 + n22) + n01 (n11 n12 + n02 (n11 + n12) + (n11 + n12) n22))
           (n03 + n23) (n22 theta22 + n23 theta23)) /
        (n11 n12 (n02 n03 n22 + n03 n22 n23 + n02 (n03 + n22) n23) +
          n01 (n03 n11 n12 n22 + (n03 n11 n12 + n11 n12 n22 + n03 (n11 + n12) n22) n23 +
              n02 (n11 + n12) (n22 n23 + n03 (n22 + n23))))}}

Out[•]= True
```

Variance computation

To compute the variance of treatment effect 2, first note

Var(theta2) = Var(w11*theta11+w12*theta12+w22*theta22+w23*theta23)

In[ ]:=
```
theta11 = n01 / (n01 + n11) * (y11 - y01);
theta12 = (n02 + n22) / (n02 + n12 + n22) * y12 -
    ((n02 / (n02 + n12 + n22)) * y02 + (n22 / (n02 + n12 + n22)) * y22);
theta22 = (n02 + n12) / (n02 + n12 + n22) * y22 -
    ((n02 / (n02 + n12 + n22)) * y02 + (n12 / (n02 + n12 + n22)) * y12);
theta23 = n03 / (n03 + n23) * (y23 - y03);
expr = w11 * theta11 + w12 * theta12 + w22 * theta22 + w23 * theta23;
Collect[FullSimplify[expr], {y01, y11, y02, y12, y22, y03, y23}];
expr01 = FullSimplify[
    (-n01 n03 n11 n12 n22 - n01 n11 n12 n22 n23) / (n01 n03 (n11 n12 + n02 (n11 + n12)) n22 +
       n01 (n03 n11 n12 + n11 n12 n22 + n03 (n11 + n12) n22 + n02 (n11 + n12) (n03 + n22)) n23 +
       n11 n12 (n02 n03 n22 + n03 n22 n23 + n02 (n03 + n22) n23))];
expr02 = FullSimplify[
    (-n01 n02 n03 n11 n22 - n01 n02 n03 n12 n22 - n02 n03 n11 n12 n22 - n01 n02 n11 n22 n23 -
       n01 n02 n12 n22 n23 - n02 n11 n12 n22 n23) / (n01 n03 (n11 n12 + n02 (n11 + n12)) n22 +
       n01 (n03 n11 n12 + n11 n12 n22 + n03 (n11 + n12) n22 + n02 (n11 + n12) (n03 + n22)) n23 +
       n11 n12 (n02 n03 n22 + n03 n22 n23 + n02 (n03 + n22) n23))];
expr03 = FullSimplify[
    (-n01 n03 n11 n12 n23 - n02 n03 n11 n12 n23 - n03 n11 n12 n22 n23 - n01 n03 n11 (n02 + n22)
        n23 - n01 n03 n12 (n02 + n22) n23) / (n01 n03 (n11 n12 + n02 (n11 + n12)) n22 +
       n01 (n03 n11 n12 + n11 n12 n22 + n03 (n11 + n12) n22 + n02 (n11 + n12) (n03 + n22)) n23 +
       n11 n12 (n02 n03 n22 + n03 n22 n23 + n02 (n03 + n22) n23))];
expr11 = FullSimplify[
    (n01 n03 n11 n12 n22 + n01 n11 n12 n22 n23) / (n01 n03 (n11 n12 + n02 (n11 + n12)) n22 +
       n01 (n03 n11 n12 + n11 n12 n22 + n03 (n11 + n12) n22 + n02 (n11 + n12) (n03 + n22)) n23 +
       n11 n12 (n02 n03 n22 + n03 n22 n23 + n02 (n03 + n22) n23))];
expr12 = FullSimplify[
    (-n01 n03 n11 n12 n22 - n01 n11 n12 n22 n23) / (n01 n03 (n11 n12 + n02 (n11 + n12)) n22 +
       n01 (n03 n11 n12 + n11 n12 n22 + n03 (n11 + n12) n22 + n02 (n11 + n12) (n03 + n22)) n23 +
       n11 n12 (n02 n03 n22 + n03 n22 n23 + n02 (n03 + n22) n23))];
expr22 = FullSimplify[
    (n02 n03 n11 n12 n22 + n01 n03 (n11 n12 + n02 (n11 + n12)) n22 + n01 n02 n12 n22 n23 + n02 n11
        n12 n22 n23 + n01 n11 (n02 + n12) n22 n23) / (n01 n03 (n11 n12 + n02 (n11 + n12)) n22 +
       n01 (n03 n11 n12 + n11 n12 n22 + n03 (n11 + n12) n22 + n02 (n11 + n12) (n03 + n22)) n23 +
       n11 n12 (n02 n03 n22 + n03 n22 n23 + n02 (n03 + n22) n23))];
expr23 = FullSimplify[ (n02 n03 n11 n12 n23 + n03 n11 n12 n22 n23 +
       n01 n03 (n11 n12 + n02 (n11 + n12) + (n11 + n12) n22) n23) /
     (n01 n03 (n11 n12 + n02 (n11 + n12)) n22 +
       n01 (n03 n11 n12 + n11 n12 n22 + n03 (n11 + n12) n22 + n02 (n11 + n12) (n03 + n22)) n23 +
       n11 n12 (n02 n03 n22 + n03 n22 n23 + n02 (n03 + n22) n23))];
FullSimplify[
  Collect[FullSimplify[expr], {y01, y11, y02, y12, y22, y03, y23}] == expr01 * y01 +
    expr02 * y02 + expr03 * y03 + expr11 * y11 + expr12 * y12 + expr22 * y22 + expr23 * y23];
```

Variance expression is then term2s*sigma^2/N, where

```
In[•]:= term2f = FullSimplify[expr01^2 * y01 + expr02^2 * y02 + expr03^2 * y03 + expr11^2 * y11 +
          expr12^2 * y12 + expr22^2 * y22 + expr23^2 * y23 /. {y01 → 1 / n01, y02 → 1 / n02,
          y03 → 1 / n03, y11 → 1 / n11, y12 → 1 / n12, y22 → 1 / n22, y23 → 1 / n23}];
        term2s =
          FullSimplify[term2f /. {n01 → r01 * Nt, n02 → r02 * Nt, n03 → r03 * Nt, n11 → r11 * Nt,
            n12 → r12 * Nt, n22 → r22 * Nt, n23 → r23 * Nt}] * Nt;
```

Define terms to optimise

```
In[•]:= substp =
          {r11 → r1 p11, r12 → r2 p12, r22 → r2 p22, r02 → r2 p02, r23 → r3 p23, r3 → 1 - r1 - r2};

In[•]:= subst = {r11 → r1 / 2 , r01 → r1 / 2 , r23 → r3 / 2 , r03 → r3 / 2, r02 → r2 - r12 - r22 };
        term1 = FullSimplify[(r11 * r01 / (r11 + r01)) + (r12 * r02 / (r12 + r02)) /. subst /. substp]
        term2 = FullSimplify[(1 / term2s) /. subst /. substp]
```

Out[•]= $\frac{r1}{4}+\frac{p12\,(-1+p12+p22)\,r2}{-1+p22}$

Out[•]= $\left(r1^2+r1\left(-1+\left(-4\,(-1+p12)\,p12+(1-2\,p22)^2\right)r2\right)+4\,p12\,r2\,(-1+p12+r2+4\,(-1+p22)\,p22\,r2+p12\,(-1+4\,p22)\,r2)\right)\big/\left(-4\,r1+16\,(-1+p12)\,p12\,r2\right)$

```
In[•]:= substg = {r01 → r1 - r11, r03 → r3 - r23, r02 → r2 - r12 - r22 };
        termg1 =
         FullSimplify[(r11 * r01 / (r11 + r01)) + (r12 * r02 / (r12 + r02)) /. substg /. substp]
        termg2 = FullSimplify[(1 / term2s) /. substg /. substp]
```

Out[•]= $p11\,(r1-p11\,r1)+\frac{p12\,(-1+p12+p22)\,r2}{-1+p22}$

Out[•]= $\frac{p22\,r2\,(-((-1+p11)\,p11\,(-1+p22)\,r1)+p12\,(-1+p12+p22)\,r2)}{(-1+p11)\,p11\,r1+(-1+p12)\,p12\,r2}+p23\,r3\left(1+\frac{p23\,r3}{-1+r1+r2}\right)$

Numerical example: optimisation assuming balanced design in periods 1 and 3

```
In[•]:= ex = {r1 → 0.1, r2 → 0.8, r3 → 0.1};
        FindMinimum[{(-term1) /. ex, term1 == term2 /. ex, p12 > 0, p22 > 0},
         {{p12, r2 / 3 /. ex}, {p22, r2 / 3 /. ex}}]

Out[•]= {-0.164091, {p12 → 0.303787, p22 → 0.289682}}
```

Optimisation (approach 1) - here we do not assume balanced design in periods 1 and 3 and thus also allocation rates in periods 1 and 3 are optimized

```
In[•]:= ex = {r1 → 0.4, r2 → 0.4, r3 → 0.2};
        FindMinimum[{(-termg1) /. ex, termg1 == termg2 /. ex,
          p12 > 0, p22 > 0, p11 > 0, p23 > 0, p11 < 1, p23 < 1},
         {{p11, r1 / 2 /. ex}, {p12, r2 / 3 /. ex}, {p22, r2 / 3 /. ex}, {p23, r3 / 2 /. ex}}]

Out[•]= {-0.144071, {p11 → 0.5, p12 → 0.153829, p22 → 0.457912, p23 → 0.5}}
```

In[ ]:=
```
FindMinimum[
  {-termg1 /. ex, termg1 == termg2 /. ex, p12 > 0, p22 > 0, p11 > 0, p23 > 0, p11 < 1, p23 < 1},
  {{p11, r1/2 /. ex}, {p12, r2/3 /. ex}, {p22, r2/3 /. ex}, {p23, r3/2 /. ex}}]
```

Out[ ]= {-0.144071, {p11 → 0.5, p12 → 0.153829, p22 → 0.457912, p23 → 0.5}}

In[ ]:=
```
termg2
```

Out[ ]= $$\frac{p22\,r2\,(-((-1+p11)\,p11\,(-1+p22)\,r1)+p12\,(-1+p12+p22)\,r2)}{(-1+p11)\,p11\,r1+(-1+p12)\,p12\,r2}+p23\,r3\left(1+\frac{p23\,r3}{-1+r1+r2}\right)$$

In[ ]:=
```
{{p11, r1/2 /. ex}, {p12, r2/3 /. ex}, {p22, r2/3 /. ex}, {p23, r3/2 /. ex}}
```

Out[ ]= {{p11, 0.2}, {p12, 0.133333}, {p22, 0.133333}, {p23, 0.1}}

Note that we cannot find analytical solutions, but the numerical solutions satisfy that the optimal design follows a balanced design in periods 1 and 3.

Optimisation (approach 2) - assume balanced designs in periods 1 and 3

In[ ]:=
```
constr = term1 - term2;
```

In[ ]:=
```
e1 = FullSimplify[Solve[D[term1, p12] == l D[constr, p12], l]]
e2 = FullSimplify[Solve[D[term1, p22] == l D[constr, p22], l]]
e3 = e1[[1]][[1]][[2]] == e2[[1]][[1]][[2]]
```

Out[ ]= $$\{\{l \to ((-1+2\,p12+p22)\,(r1-4\,(-1+p12)\,p12\,r2)^2) / ((-1+2\,p12+p22)\,r1^2-8\,p12\,(2\,p12^2+p12\,(-3+p22)-(-1+p22)\,(1+p22^2))\,r1\,r2+16\,p12^2\,(-1+p12+p22)\,(1+2\,p12^2+p22^2-p12\,(3+p22))\,r2^2)\}\}$$

Out[ ]= $$\{\{l \to (p12^2\,(-r1+4\,(-1+p12)\,p12\,r2)) / (-p12^2\,r1+(-1+p22)^2\,(-1+2\,p22)\,r1+4\,p12\,(-1+p12+p22)\,(1+p12^2-(3+p12)\,p22+2\,p22^2)\,r2)\}\}$$

Out[ ]= $$((-1+2\,p12+p22)\,(r1-4\,(-1+p12)\,p12\,r2)^2) / ((-1+2\,p12+p22)\,r1^2-8\,p12\,(2\,p12^2+p12\,(-3+p22)-(-1+p22)\,(1+p22^2))\,r1\,r2+16\,p12^2\,(-1+p12+p22)\,(1+2\,p12^2+p22^2-p12\,(3+p22))\,r2^2) == (p12^2\,(-r1+4\,(-1+p12)\,p12\,r2)) / (-p12^2\,r1+(-1+p22)^2\,(-1+2\,p22)\,r1+4\,p12\,(-1+p12+p22)\,(1+p12^2-(3+p12)\,p22+2\,p22^2)\,r2)$$

In[ ]:=
```
sol2 = Solve[e3, {p12}];
```

In[ ]:=
```
solsim = Simplify[sol2[[7]]]
```

Out[ ]=
```
{p12 →
   1/(24 (-1 + p22) r2) (-4 (3 - 6 p22 + 2 p22^2) r2 + (2 × 2^(1/3) (1 - i √3) r2 ((3 - 9 p22 + 6 p22^2) r1 +
          (3 - 12 p22 + 18 p22^2 - 12 p22^3 + 4 p22^4) r2)) / (-9 p22^2 r1 r2^2 + 27 p22^3 r1 r2^2 -
          18 p22^4 r1 r2^2 + 18 p22^2 r2^3 - 72 p22^3 r2^3 + 108 p22^4 r2^3 - 72 p22^5 r2^3 + 16 p22^6 r2^3 +
          √(r2^3 (-4 ((3 - 9 p22 + 6 p22^2) r1 + (3 - 12 p22 + 18 p22^2 - 12 p22^3 + 4 p22^4) r2)^3 +
              p22^4 r2 (9 (1 - 3 p22 + 2 p22^2) r1 -
                  2 (9 - 36 p22 + 54 p22^2 - 36 p22^3 + 8 p22^4) r2)^2)))^(1/3) +
      2^(2/3) (1 + i √3) (-9 p22^2 r1 r2^2 + 27 p22^3 r1 r2^2 - 18 p22^4 r1 r2^2 + 18 p22^2 r2^3 -
          72 p22^3 r2^3 + 108 p22^4 r2^3 - 72 p22^5 r2^3 + 16 p22^6 r2^3 +
          √(r2^3 (-4 ((3 - 9 p22 + 6 p22^2) r1 + (3 - 12 p22 + 18 p22^2 - 12 p22^3 + 4 p22^4) r2)^3 +
              p22^4 r2 (9 (1 - 3 p22 + 2 p22^2) r1 -
                  2 (9 - 36 p22 + 54 p22^2 - 36 p22^3 + 8 p22^4) r2)^2)))^(1/3))}
```

In[ ]:=
```
$Assumptions = p12 > 0 && p22 > 0 && Element[p12, Reals] && Element[p22, Reals]
```

Out[ ]=
```
p12 > 0 && p22 > 0 && p12 ∈ ℝ && p22 ∈ ℝ
```

In[ ]:=
```
Re[solsim]
```

Out[ ]=
```
{Re[p12 →
   1/(24 (-1 + p22) r2) (-4 (3 - 6 p22 + 2 p22^2) r2 + (2 × 2^(1/3) (1 - i √3) r2 ((3 - 9 p22 + 6 p22^2) r1 +
          (3 - 12 p22 + 18 p22^2 - 12 p22^3 + 4 p22^4) r2)) / (-9 p22^2 r1 r2^2 + 27 p22^3 r1 r2^2 -
          18 p22^4 r1 r2^2 + 18 p22^2 r2^3 - 72 p22^3 r2^3 + 108 p22^4 r2^3 - 72 p22^5 r2^3 + 16 p22^6 r2^3 +
          √(r2^3 (-4 ((3 - 9 p22 + 6 p22^2) r1 + (3 - 12 p22 + 18 p22^2 - 12 p22^3 + 4 p22^4) r2)^3 +
              p22^4 r2 (9 (1 - 3 p22 + 2 p22^2) r1 -
                  2 (9 - 36 p22 + 54 p22^2 - 36 p22^3 + 8 p22^4) r2)^2)))^(1/3) +
      2^(2/3) (1 + i √3) (-9 p22^2 r1 r2^2 + 27 p22^3 r1 r2^2 - 18 p22^4 r1 r2^2 + 18 p22^2 r2^3 -
          72 p22^3 r2^3 + 108 p22^4 r2^3 - 72 p22^5 r2^3 + 16 p22^6 r2^3 +
          √(r2^3 (-4 ((3 - 9 p22 + 6 p22^2) r1 + (3 - 12 p22 + 18 p22^2 - 12 p22^3 + 4 p22^4) r2)^3 +
              p22^4 r2 (9 (1 - 3 p22 + 2 p22^2) r1 -
                  2 (9 - 36 p22 + 54 p22^2 - 36 p22^3 + 8 p22^4) r2)^2)))^(1/3))]}
```

In[ ]:=
```
sol2 /. ex /. p22 → 0.23 / 0.8
```

Out[ ]=
```
{{p12 → -0.207107}, {p12 → 1.20711}, {p12 → -0.196053},
 {p12 → 0.908553}, {p12 → 0.866963 - 5.55112 × 10^-17 i},
 {p12 → -0.185879 - 2.77556 × 10^-17 i}, {p12 → 0.329662 + 1.11022 × 10^-16 i}}
```

In[ ]:= **eq = FullSimplify[term1 - term2]**

Out[ ]= $$\frac{1}{4}\left(r1+\frac{4\,p12\,(-1+p12+p22)\,r2}{-1+p22}+\frac{1}{r1-4\,(-1+p12)\,p12\,r2}\left(r1^2+r1\left(-1+\left(-4\,(-1+p12)\,p12+(1-2\,p22)^2\right)r2\right)+4\,p12\,r2\,(-1+p12+r2+4\,(-1+p22)\,p22\,r2+p12\,(-1+4\,p22)\,r2)\right)\right)$$

In[ ]:= **eq3 = FullSimplify[e3]**

Out[ ]= $$(r1-4\,(-1+p12)\,p12\,r2)\left(p12^2\Big/\left(-p12^2\,r1+(-1+p22)^2\,(-1+2\,p22)\,r1+4\,p12\,(-1+p12+p22)\left(1+p12^2-(3+p12)\,p22+2\,p22^2\right)r2\right)+((-1+2\,p12+p22)\,(r1-4\,(-1+p12)\,p12\,r2))\Big/\left((-1+2\,p12+p22)\,r1^2-8\,p12\left(2\,p12^2+p12\,(-3+p22)-(-1+p22)\left(1+p22^2\right)\right)r1\,r2+16\,p12^2\,(-1+p12+p22)\left(1+2\,p12^2+p22^2-p12\,(3+p22)\right)r2^2\right)\right)==0$$

In[ ]:= **NSolve[{eq == 0 /. ex, eq3 /. ex}, {p12, p22}]**

Out[ ]= {{p22 → -28 924.1 + 18 543.2 i, p12 → -0.249998 + 9.81706 × 10^{-7} i},
{p22 → -0.0311394 + 0.265623 i, p12 → -0.211592 + 0.0051594 i},
{p22 → -0.0311394 - 0.265623 i, p12 → -0.211592 - 0.0051594 i},
{p22 → -0.0311394 + 0.265623 i, p12 → -0.211592 + 0.0051594 i},
{p22 → 0.457912, p12 → 0.153829}, {p22 → 0.457912, p12 → 0.153829},
{p22 → 0.7153 + 0.205041 i, p12 → 0.151828 - 0.155807 i},
{p22 → 0.7153 - 0.205041 i, p12 → 0.151828 + 0.155807 i},
{p22 → 1.84182, p12 → 1.94225}, {p22 → 0.5, p12 → 0.5}, {p22 → 0.5, p12 → 0.5},
{p22 → 0.117486 - 0.930047 i, p12 → 1.17003 + 0.720353 i},
{p22 → 0.117486 + 0.930047 i, p12 → 1.17003 - 0.720353 i},
{p22 → 1.59697, p12 → -0.816608}}

Supplementary Material of “Optimal allocation strategies in platform trials”

Optimisation under unequal variances

Variance estimator of effect 1 under unequal variances

In[ ]:=
```
w11 = 1 / s11^2 / ( 1 / s11^2 + 1 / s12^2)
```

Out[ ]=
$$\frac{1}{s11^2\left(\frac{1}{s11^2}+\frac{1}{s12^2}\right)}$$

In[ ]:=
```
w12 = 1 / s12^2 / ( 1 / s11^2 + 1 / s12^2)
```

Out[ ]=
$$\frac{1}{\left(\frac{1}{s11^2}+\frac{1}{s12^2}\right)s12^2}$$

In[ ]:=
```
s11 = Sqrt[s0^2 * (rs10^2 / n11 + 1 / n01)]
```

Out[ ]=
$$\sqrt{\left(\frac{1}{n01}+\frac{rs10^2}{n11}\right)s0^2}$$

In[ ]:=
```
s12 = Sqrt[s0^2 * (rs10^2 / n12 + 1 / n02)]
```

Out[ ]=
$$\sqrt{\left(\frac{1}{n02}+\frac{rs10^2}{n12}\right)s0^2}$$

In[ ]:=
```
var1 = w11^2 * s11^2 + w12^2 * s12^2
```

Out[ ]=
$$\frac{1}{\left(\frac{1}{n01}+\frac{rs10^2}{n11}\right)\left(\frac{1}{\left(\frac{1}{n01}+\frac{rs10^2}{n11}\right)s0^2}+\frac{1}{\left(\frac{1}{n02}+\frac{rs10^2}{n12}\right)s0^2}\right)^2 s0^2}+\frac{1}{\left(\frac{1}{n02}+\frac{rs10^2}{n12}\right)\left(\frac{1}{\left(\frac{1}{n01}+\frac{rs10^2}{n11}\right)s0^2}+\frac{1}{\left(\frac{1}{n02}+\frac{rs10^2}{n12}\right)s0^2}\right)^2 s0^2}$$

In[ ]:=
```
FullSimplify[var1]
```

Out[ ]=
$$\frac{\left(n11+n01\,rs10^2\right)\left(n12+n02\,rs10^2\right)s0^2}{(n01+n02)\,n11\,n12+n01\,n02\,(n11+n12)\,rs10^2}$$

In[ ]:=
```
subst = {n11 → r1 * N / 2 , n01 → r1 * N / 2 ,
   n12 → r2 * N - n02 - n22 , n03 → r3 * N / 2, n23 → r3 * N / 2};
substp = {n12 → r2 * N * p12, n22 → r2 * N * p22, n02 → r2 * N * p02, r3 → 1 - r1 - r2};
example = {r1 → .2, r2 → .6, rs10 → 1.5, rs20 → 1, p02 → 0.41421356237309515`};
```

In[ ]:=
```
invvar1 = Simplify[1 / (var1)]
```

Out[ ]=
$$\frac{n02\,n11\,n12+n01\,n02\,n12\,rs10^2+n01\,n11\left(n12+n02\,rs10^2\right)}{\left(n11+n01\,rs10^2\right)\left(n12+n02\,rs10^2\right)s0^2}$$

In[∘]:=
```
term1uneq = FullSimplify[invvar1 / (N / s0^2) /. subst /. substp]
```

Out[∘]= $\frac{r1}{2+2\,rs10^2}-\frac{p02\,(-1+p02+p22)\,r2}{1-p22+p02\,(-1+rs10^2)}$

In[∘]:=
```
eqvar1 = FullSimplify[var1 /. subst /. substp]
```

Out[∘]= $-\frac{2\,(1+rs10^2)\,(1-p22+p02\,(-1+rs10^2))\,s0^2}{N\,(2\,p02\,(-1+p02+p22)\,r2\,(1+rs10^2)+r1\,(-1+p02+p22-p02\,rs10^2))}$

In[∘]:=
```
(*Check case with equal variances*)
```

In[∘]:=
```
Simplify[term1uneq /. {rs10 → 1, N → 1, s0 → 1}]
```

Out[∘]= $\frac{r1}{4}+\frac{p02\,(-1+p02+p22)\,r2}{-1+p22}$

Variance estimator of effect 2 under unequal variances

In[∘]:=
```
w22 = 1 / s22^2 / (1 / s22^2 + 1 / s23^2);
w23 = 1 / s23^2 / (1 / s22^2 + 1 / s23^2);

s22 = Sqrt[s0^2 * (rs20^2 / n22 + 1 / n02)];
s23 = Sqrt[s0^2 * (rs20^2 / n23 + 1 / n03)];
```

In[∘]:=
```
var2 = w22^2 * s22^2 + w23^2 * s23^2
```

Out[∘]= $\frac{1}{\left(\frac{1}{n02}+\frac{rs20^2}{n22}\right)\left(\frac{1}{\left(\frac{1}{n02}+\frac{rs20^2}{n22}\right)s0^2}+\frac{1}{\left(\frac{1}{n03}+\frac{rs20^2}{n23}\right)s0^2}\right)^2 s0^2}+\frac{1}{\left(\frac{1}{n03}+\frac{rs20^2}{n23}\right)\left(\frac{1}{\left(\frac{1}{n02}+\frac{rs20^2}{n22}\right)s0^2}+\frac{1}{\left(\frac{1}{n03}+\frac{rs20^2}{n23}\right)s0^2}\right)^2 s0^2}$

In[∘]:=
```
invvar2 = Simplify[1 / (var2)]
```

Out[∘]= $\frac{n03\,n22\,n23+n02\,n03\,n23\,rs20^2+n02\,n22\,(n23+n03\,rs20^2)}{(n22+n02\,rs20^2)\,(n23+n03\,rs20^2)\,s0^2}$

In[∘]:=
```
term2uneq = FullSimplify[invvar2 / (N / s0^2) /. subst /. substp]
```

Out[∘]= $-\frac{-1+r1+r2}{2\,(1+rs20^2)}+\frac{p02\,p22\,r2}{p22+p02\,rs20^2}$

In[∘]:=
```
(*Check case equal variances*)
```

In[∘]:=
```
FullSimplify[term2uneq /. {rs20 → 1, N → 1, s0 → 1}]
```

Out[∘]= $\frac{1}{4}\,(1-r1-r2)+\frac{p02\,p22\,r2}{p02+p22}$

In[∘]:=
```
eqvar2 = FullSimplify[var2 /. subst /. substp]
```

Out[∘]= $-\frac{2\,(1+rs20^2)\,(p22+p02\,rs20^2)\,s0^2}{N\,p22\,(-1+r1+r2-2\,p02\,r2)+N\,p02\,(-1+r1+r2-2\,p22\,r2)\,rs20^2}$

Optimisation

In[∘]:=
```
(*Constraint equal variances between treatment effect estimators*)
```

In[ ]:=
```
constr = FullSimplify[term1uneq - term2uneq]
```

Out[ ]=
$$\frac{r1}{2+2\,rs10^2}-\frac{p02\,(-1+p02+p22)\,r2}{1-p22+p02\,(-1+rs10^2)}+\frac{-1+r1+r2}{2\,(1+rs20^2)}-\frac{p02\,p22\,r2}{p22+p02\,rs20^2}$$

In[ ]:=
```
Simplify[(eqvar1 - eqvar2) * (N / s0^2)]
```

Out[ ]=
$$-\frac{2\,(1+rs10^2)\,(1-p22+p02\,(-1+rs10^2))}{2\,p02\,(-1+p02+p22)\,r2\,(1+rs10^2)+r1\,(-1+p02+p22-p02\,rs10^2)}+\frac{2\,N\,(1+rs20^2)\,(p22+p02\,rs20^2)}{N\,p22\,(-1+r1+r2-2\,p02\,r2)+N\,p02\,(-1+r1+r2-2\,p22\,r2)\,rs20^2}$$

In[ ]:=
```
Solve[Simplify[term1uneq - term2uneq] == 0, p22] /. example
```

Out[ ]=
```
{{p22 → 0.136517}, {p22 → 18.3645}}
```

In[ ]:=
```
FullSimplify[Solve[Simplify[term1uneq - term2uneq] == 0, p22][[2]]]
```

Out[ ]=
$$\Big\{p22\to\frac{1}{2\,((-1+r2)\,(1+rs10^2)+r1\,(2+rs10^2+rs20^2))}\Big(-1+2\,r1+r2-rs10^2+r1\,rs10^2+r2\,rs10^2+r1\,rs20^2-2\,p02^2\,r2\,(1+rs10^2)\,(1+rs20^2)\,(rs10^2+rs20^2)+p02\,(-1+rs10^2-rs20^2)\,((-1+r2)\,(1+rs10^2)+r1\,(2+rs10^2+rs20^2))-\sqrt{\Big(((-1+r2)\,(1+rs10^2)-2\,p02^2\,r2\,(1+rs10^2)\,(1+rs20^2)\,(rs10^2+rs20^2)+r1\,(2+rs10^2+rs20^2)+p02\,(-1+rs10^2-rs20^2)\,((-1+r2)\,(1+rs10^2)+r1\,(2+rs10^2+rs20^2)))^2+4\,p02\,rs20^2\,((-1+r2)\,(1+rs10^2)+r1\,(2+rs10^2+rs20^2))\,((-1+r2)\,(1+rs10^2)-2\,p02^2\,r2\,(1+rs10^2)\,(1+rs20^2)+r1\,(2+rs10^2+rs20^2)+p02\,r1\,(-1+rs10^2)\,(2+rs10^2+rs20^2)+p02\,(1+rs10^2)\,(1-rs10^2+r2\,(1+rs10^2+2\,rs20^2)))\Big)}\Big)\Big\}$$

In[ ]:=
```
solp22 = FullSimplify[Solve[Simplify[term1uneq - term2uneq] == 0, p22][[2]]]
```

Out[ ]=
$$\Big\{p22\to\frac{1}{2\,((-1+r2)\,(1+rs10^2)+r1\,(2+rs10^2+rs20^2))}\Big(-1+2\,r1+r2-rs10^2+r1\,rs10^2+r2\,rs10^2+r1\,rs20^2-2\,p02^2\,r2\,(1+rs10^2)\,(1+rs20^2)\,(rs10^2+rs20^2)+p02\,(-1+rs10^2-rs20^2)\,((-1+r2)\,(1+rs10^2)+r1\,(2+rs10^2+rs20^2))-\sqrt{\Big(((-1+r2)\,(1+rs10^2)-2\,p02^2\,r2\,(1+rs10^2)\,(1+rs20^2)\,(rs10^2+rs20^2)+r1\,(2+rs10^2+rs20^2)+p02\,(-1+rs10^2-rs20^2)\,((-1+r2)\,(1+rs10^2)+r1\,(2+rs10^2+rs20^2)))^2+4\,p02\,rs20^2\,((-1+r2)\,(1+rs10^2)+r1\,(2+rs10^2+rs20^2))\,((-1+r2)\,(1+rs10^2)-2\,p02^2\,r2\,(1+rs10^2)\,(1+rs20^2)+r1\,(2+rs10^2+rs20^2)+p02\,r1\,(-1+rs10^2)\,(2+rs10^2+rs20^2)+p02\,(1+rs10^2)\,(1-rs10^2+r2\,(1+rs10^2+2\,rs20^2)))\Big)}\Big)\Big\}$$

In[ ]:=
```
term1uneq
```

Out[ ]=
$$\frac{r1}{2+2\,rs10^2}-\frac{p02\,(-1+p02+p22)\,r2}{1-p22+p02\,(-1+rs10^2)}$$

In[∘]:= **term2uneq**

Out[∘]=
$$-\frac{-1+r1+r2}{2\left(1+rs20^2\right)}+\frac{p02\ p22\ r2}{p22+p02\ rs20^2}$$

In[∘]:= **term1subs = FullSimplify[term1uneq /. solp22]**

Out[∘]=
$$\Big(-\left(\left(-1+(-1+r1)\ rs10^2-r1\ rs20^2\right)\left(1+p02\left(-1+rs10^2+rs20^2\right)\right)\right)+r2\left(1+rs10^2\right)\left(-1+p02\left(5-rs10^2+3\ rs20^2+2\ p02\left(1+rs20^2\right)\left(-2+rs10^2+rs20^2\right)\right)\right)+\sqrt{\Big(\Big((-1+r2)\left(1+rs10^2\right)-2\ p02^2\ r2\left(1+rs10^2\right)\left(1+rs20^2\right)\left(rs10^2+rs20^2\right)+r1\left(2+rs10^2+rs20^2\right)+p02\left(-1+rs10^2-rs20^2\right)\left((-1+r2)\left(1+rs10^2\right)+r1\left(2+rs10^2+rs20^2\right)\right)\Big)^2+4\ p02\ rs20^2\left((-1+r2)\left(1+rs10^2\right)+r1\left(2+rs10^2+rs20^2\right)\right)\Big((-1+r2)\left(1+rs10^2\right)-2\ p02^2\ r2\left(1+rs10^2\right)\left(1+rs20^2\right)+r1\left(2+rs10^2+rs20^2\right)+p02\ r1\left(-1+rs10^2\right)\left(2+rs10^2+rs20^2\right)+p02\left(1+rs10^2\right)\left(1-rs10^2+r2\left(1+rs10^2+2\ rs20^2\right)\right)\Big)\Big)}\Big)\Big/\left(4\left(1+rs10^2\right)\left(1+rs20^2\right)\left(1+p02\left(-1+rs10^2+rs20^2\right)\right)\right)$$

In[∘]:= **term2subs = FullSimplify[term2uneq /. solp22]**

Out[∘]=
$$\Big(-\left(\left(-1+(-1+r1)\ rs10^2-r1\ rs20^2\right)\left(1+p02\left(-1+rs10^2+rs20^2\right)\right)\right)+r2\left(1+rs10^2\right)\left(-1+p02\left(5-rs10^2+3\ rs20^2+2\ p02\left(1+rs20^2\right)\left(-2+rs10^2+rs20^2\right)\right)\right)+\sqrt{\Big(\Big((-1+r2)\left(1+rs10^2\right)-2\ p02^2\ r2\left(1+rs10^2\right)\left(1+rs20^2\right)\left(rs10^2+rs20^2\right)+r1\left(2+rs10^2+rs20^2\right)+p02\left(-1+rs10^2-rs20^2\right)\left((-1+r2)\left(1+rs10^2\right)+r1\left(2+rs10^2+rs20^2\right)\right)\Big)^2+4\ p02\ rs20^2\left((-1+r2)\left(1+rs10^2\right)+r1\left(2+rs10^2+rs20^2\right)\right)\Big((-1+r2)\left(1+rs10^2\right)-2\ p02^2\ r2\left(1+rs10^2\right)\left(1+rs20^2\right)+r1\left(2+rs10^2+rs20^2\right)+p02\ r1\left(-1+rs10^2\right)\left(2+rs10^2+rs20^2\right)+p02\left(1+rs10^2\right)\left(1-rs10^2+r2\left(1+rs10^2+2\ rs20^2\right)\right)\Big)\Big)}\Big)\Big/\left(4\left(1+rs10^2\right)\left(1+rs20^2\right)\left(1+p02\left(-1+rs10^2+rs20^2\right)\right)\right)$$

In[∘]:= **dfdp02 = D[term1uneq, p02]**

Out[∘]=
$$\frac{p02\ (-1+p02+p22)\ r2\left(-1+rs10^2\right)}{\left(1-p22+p02\left(-1+rs10^2\right)\right)^2}-\frac{p02\ r2}{1-p22+p02\left(-1+rs10^2\right)}-\frac{(-1+p02+p22)\ r2}{1-p22+p02\left(-1+rs10^2\right)}$$

In[∘]:= **dfdp02s = Simplify[dfdp02]**

Out[∘]=
$$\frac{r2\left(2\ p02\ (-1+p22)+(-1+p22)^2-p02^2\left(-1+rs10^2\right)\right)}{\left(-1+p02+p22-p02\ rs10^2\right)^2}$$

In[∘]:= **solp02 = Solve[dfdp02s == 0, p02]**

Out[∘]=
$$\left\{\left\{p02\to\frac{-1+p22}{-1+rs10}\right\},\left\{p02\to\frac{1-p22}{1+rs10}\right\}\right\}$$

In[∘]:= **solp02⟦2⟧ /. {p22 → .3, rs10 → 1}**

Out[∘]= $\{p02\to 0.35\}$

Supplementary Material of “Optimal allocation strategies in platform trials”

Optimisation of the sum of variances

In[ ]:=
```
subst = {n11 → r1 * (1 - p01) * N , n01 → r1 * p01 * N,
    n12 → r2 * N - n02 - n22 , n03 → r3 * p03 * N, n23 → r3 * (1 - p03) * N};
substp = {n12 → r2 * N * p12, n22 → r2 * N * p22, n02 → r2 * N * p02, r3 → 1 - r1 - r2};
```

In[ ]:=
```
term1 =
 FullSimplify[((n11 * n01 / (n11 + n01)) + (n12 * n02 / (n12 + n02))) / N /. subst /. substp]
term2 =
 FullSimplify[(n22 * n02 / (n22 + n02) / N) + (n23 * n03 / (n23 + n03) / N) /. subst /. substp]
```

Out[ ]=
$$-((-1+p01)\,p01\,r1)+\frac{p02\,(-1+p02+p22)\,r2}{-1+p22}$$

Out[ ]=
$$\frac{(-1+p03)\,p03\,(p02+p22)\,(-1+r1)+(-1+p03)\,p03\,p22\,r2+p02\,((-1+p03)\,p03+p22)\,r2}{p02+p22}$$

In[ ]:=
```
f[p02_, p22_, p01_, p03_, r2_] := FullSimplify[term1 + term2]
```

In[ ]:=
```
(*Set constraints*)
constraints = 0 ≤ p02 ≤ 1 && 0 ≤ p22 ≤ 1 && 0 ≤ p01 ≤ 1 && 0 ≤ p03 ≤ 1 && r2 > 0 && r1 > 0
(*Calculate the derivatives*)
dfdp02 = D[f[p02, p22, p01, p03, r2], p02]
dfdp22 = D[f[p02, p22, p01, p03, r2], p22]
dfdp01 = D[f[p02, p22, p01, p03, r2], p01]
dfdp03 = D[f[p02, p22, p01, p03, r2], p03]
```

Out[ ]=
$$0\le p02\le 1\,\&\&\,0\le p22\le 1\,\&\&\,0\le p01\le 1\,\&\&\,0\le p03\le 1\,\&\&\,r2>0\,\&\&\,r1>0$$

Out[ ]=
$$\frac{p02\,r2}{-1+p22}+\frac{(-1+p02+p22)\,r2}{-1+p22}+\frac{(-1+p03)\,p03\,(-1+r1)+((-1+p03)\,p03+p22)\,r2}{p02+p22}-\frac{(-1+p03)\,p03\,(p02+p22)\,(-1+r1)+(-1+p03)\,p03\,p22\,r2+p02\,((-1+p03)\,p03+p22)\,r2}{(p02+p22)^2}$$

Out[ ]=
$$\frac{p02\,r2}{-1+p22}-\frac{p02\,(-1+p02+p22)\,r2}{(-1+p22)^2}+\frac{(-1+p03)\,p03\,(-1+r1)+p02\,r2+(-1+p03)\,p03\,r2}{p02+p22}-\frac{(-1+p03)\,p03\,(p02+p22)\,(-1+r1)+(-1+p03)\,p03\,p22\,r2+p02\,((-1+p03)\,p03+p22)\,r2}{(p02+p22)^2}$$

Out[ ]=
$$-((-1+p01)\,r1)-p01\,r1$$

Out[ ]=
$$\frac{1}{p02+p22}\big((-1+p03)\,(p02+p22)\,(-1+r1)+p03\,(p02+p22)\,(-1+r1)+p02\,(-1+2\,p03)\,r2+(-1+p03)\,p22\,r2+p03\,p22\,r2\big)$$

In[ ]:=
```
dfdp02s = Simplify[dfdp02]
```

Out[ ]=
$$\frac{\left(2\,p02^3+2\,(-1+p22)\,p22^2+2\,p02\,p22\,(-1+2\,p22)+p02^2\,(-1+5\,p22)\right)r2}{(-1+p22)\,(p02+p22)^2}$$

In[ ]:=
```
dfdp22s = Simplify[dfdp22]
```

Out[ ]=
$$-\frac{p02^2\,(1+p02)\,(-1+p02+2\,p22)\,r2}{(-1+p22)^2\,(p02+p22)^2}$$

In[ ]:=
```
dfdp01s = Simplify[dfdp01]
```

Out[ ]= $r1 - 2\,p01\,r1$

In[ ]:=
```
dfdp03s = Simplify[dfdp03]
```

Out[ ]= $(-1+2\,p03)\,(-1+r1+r2)$

In[ ]:=
```
(*Solve for critical points*)
criticalPoints = NSolve[
  {dfdp02s == 0, dfdp22s == 0, dfdp01s == 0, dfdp03s == 0, constraints}, {p02, p22, p01, p03}]
```

Out[ ]=
$$\left\{\left\{p02 \to 0.414214 \text{ if } r2>0 \,\&\&\, r1>0,\ p22 \to 0.292893 \text{ if } r2>0 \,\&\&\, r1>0,\ p01 \to 0.5 \text{ if } r2>0 \,\&\&\, r1>0,\ p03 \to \frac{-1.+r1+r2}{-2.+2.\,r1+2.\,r2} \text{ if } r2>0 \,\&\&\, r1>0\right\}\right\}$$

In[ ]:=
```
(*Evaluate the function at critical points*)
min = MinimalBy[criticalPoints, f[p02, p22, r2] /. # &]
```

Out[ ]=
$$\left\{\left\{p02 \to 0.414214 \text{ if } r2>0 \,\&\&\, r1>0,\ p22 \to 0.292893 \text{ if } r2>0 \,\&\&\, r1>0,\ p01 \to 0.5 \text{ if } r2>0 \,\&\&\, r1>0,\ p03 \to \frac{-1.+r1+r2}{-2.+2.\,r1+2.\,r2} \text{ if } r2>0 \,\&\&\, r1>0\right\}\right\}$$

In[ ]:=
```
Solve[{dfdp02s == 0, dfdp22s == 0, dfdp01s == 0, dfdp03s == 0, constraints},
 {p02, p22, p01, p03}]
```

Out[ ]=
$$\left\{\left\{p02 \to -1+\sqrt{2} \text{ if } r2>0 \,\&\&\, r1>0,\ p22 \to \frac{-\left(-1+\sqrt{2}\right)^2 r2+\left(-1+\sqrt{2}\right)^4 r2}{-2\left(-1+\sqrt{2}\right)^2 r2-2\left(-1+\sqrt{2}\right)^3 r2} \text{ if } r2>0 \,\&\&\, r1>0,\ p01 \to \frac{1}{2} \text{ if } r2>0 \,\&\&\, r1>0,\ p03 \to \frac{-1+r1+r2}{-2+2\,r1+2\,r2} \text{ if } r2>0 \,\&\&\, r1>0\right\}\right\}$$
